# A HAT7/ATHB13/brassinosteroid signaling module mediates growth of laticifer cells in *Euphorbia lathyris*

**DOI:** 10.1093/plcell/koag289

**Published:** 2026-10-17

**Authors:** Francisco J Escaray, Cristian J Antonelli, Begoña Balaguer, Carlos Vera-Antón, Joaquín Grau, José M Franco-Zorrilla, Pablo Vera

**Affiliations:** Instituto de Biología Molecular y Celular de Plantas, Universidad Politécnica de Valencia-Consejo Superior de Investigaciones Científicas, Ciudad Politécnica de la Innovación, Edificio 8E, acceso G, Ingeniero Fausto Elio, s/n, Valencia 46022, Spain; Instituto de Biología Molecular y Celular de Plantas, Universidad Politécnica de Valencia-Consejo Superior de Investigaciones Científicas, Ciudad Politécnica de la Innovación, Edificio 8E, acceso G, Ingeniero Fausto Elio, s/n, Valencia 46022, Spain; On leaving from Instituto de Fisiología Vegetal (INFIVE), Universidad Nacional de La Plata (UNLP), Consejo Nacional de Investigaciones Científicas y Técnicas (CONICET), La Plata, Argentina; Instituto de Biología Molecular y Celular de Plantas, Universidad Politécnica de Valencia-Consejo Superior de Investigaciones Científicas, Ciudad Politécnica de la Innovación, Edificio 8E, acceso G, Ingeniero Fausto Elio, s/n, Valencia 46022, Spain; Instituto de Biología Molecular y Celular de Plantas, Universidad Politécnica de Valencia-Consejo Superior de Investigaciones Científicas, Ciudad Politécnica de la Innovación, Edificio 8E, acceso G, Ingeniero Fausto Elio, s/n, Valencia 46022, Spain; Department of Plant Molecular Genetics, Centro Nacional de Biotecnología (CNB)-Consejo Superior de Investigaciones Científicas (CSIC), Darwin 3, Madrid 28049, Spain; Department of Plant Molecular Genetics, Centro Nacional de Biotecnología (CNB)-Consejo Superior de Investigaciones Científicas (CSIC), Darwin 3, Madrid 28049, Spain; Instituto de Biología Molecular y Celular de Plantas, Universidad Politécnica de Valencia-Consejo Superior de Investigaciones Científicas, Ciudad Politécnica de la Innovación, Edificio 8E, acceso G, Ingeniero Fausto Elio, s/n, Valencia 46022, Spain

## Abstract

Laticifers, the coenocytic cells responsible for latex production, represent one of the most extreme examples of continuous and polarized growth in plants. Their unique architecture, extending over large distances, requires tight coordination between wall extensibility, cytoplasmic organization, and hormonal control. Here, we characterized the *poor in latex 13* (*pil13*) mutant of *Euphorbia lathyrism*, which, similar to the previously described *pil1* and *pil6* mutants, is defective in laticifer expansion during post-embryonic phases of plant growth. Through comparative transcriptomic analyses of these 3 *pil* mutants, we identified a core set of 266 differentially expressed genes (DEGs) that define a transcriptomic signature associated with laticifers. We further identified a regulatory network linking 2 HD-Zip I transcription factors, HAT7 and ATHB13, with brassinosteroid (BR) signaling in the mediation of laticifer growth. This HAT7/ATHB13–BR module regulates the expression of laticifer DEGs involved in cell wall remodeling. Consequently, we found that laticifer cell walls are highly enriched in low-esterified homogalacturonan (HG) and branched rhamnogalacturonan-I (RG-I) pectins. Whole-mount immunolocalization revealed that laticifer cell walls exhibit a peculiar spatial segregation of pectic polysaccharides. These pectins follow a characteristic complementary, non-overlapping deposition pattern along the laticifer longitudinal axis, presumably conferring both elasticity and mechanical integrity. This modular pectin assembly appears compatible with the high flexibility and mechanical sinuosity required for growing laticifers through host tissues, while accommodating internal turgor pressure changes derived from latex accumulation. Our findings define the laticifer as a cellular model in which wall remodeling, cell growth, turgor pressure, and hormonal signaling are mechanistically coupled.

## Introduction

Understanding cell differentiation and specialization is fundamental to developmental biology (Casey et al. 2020). In plants, cell fate results from complex interactions between transcriptional networks, hormonal signaling, and intercellular communication (Ryu et al. 2021; Rusnak et al. 2024). Consequently, during development, various tissue types communicate to ensure proper spatial organization of cell types (Poethig 1989), often occurring concurrently with cell growth and division. Furthermore, mechanical forces also influence cell identity and morphogenesis (Iida et al. 2023). Since plant cells have rigid cell walls, fate-specification programs depend on the composition and mechanical properties of this extracellular compartment. Thus, biosynthetic and modifying enzymes involved in cell wall formation must be tightly regulated by gene regulatory networks driving morphogenesis (Peaucelle et al. 2008, 2011; Qi et al. 2017; Qiu et al. 2021). Beyond its structural role, the cell wall integrates developmental and mechanical information, and its capacity to balance strength and extensibility relies on remodeling of its major polymers—cellulose, hemicellulose, and pectins—(Anderson and Kieber 2020). Among these, pectins are particularly dynamic and influence wall rigidity and the capacity for anisotropic expansion (Mohnen 2008). Recent studies show that receptor-like kinases (eg, RLP44, FERONIA, WAKs) sense pectin structure to monitor wall integrity and activate brassinosteroid (BR) hormone signaling for wall remodeling (Kohorn et al. 2009; Wolf et al. 2014; Lin et al. 2022; Huerta et al. 2023). These findings highlight the integration of cell wall dynamics and hormonal control as a core axis of plant cell differentiation.

Laticifers provide an exceptional model to study how signaling, transcriptional, and mechanical systems converge to generate specialized cell identities. These secretory cells synthesize and store latex—a complex emulsion rich in isoprenoids, proteins, and defense metabolites—exuded upon injury to protect plants from herbivory (Dussourd and Eisner 1987; Agrawal and Konno 2009; Castelblanque et al. 2021). Laticifers occur in at least 41 angiosperm families and have evolved independently multiple times, reflecting a significant adaptive advantage (Metcalfe 1967; Tomlinson and Fisher 2005). Despite their ecological importance and evolutionary significance, the molecular mechanisms driving laticifer specification, growth, and maintenance remain poorly understood. Non-articulated laticifers, which are highly elongated multinucleated cells, are particularly abundant in the Euphorbiaceae family. These cells originate during early embryogenesis, at the globular stage, through a pair of symmetric differentiation events that produce laticifer initials (Mahlberg 1963; Mahlberg and Sabharwal 1968; Castelblanque et al. 2016). As the embryo develops, these initials elongate and undergo successive nuclear divisions without cytokinesis, forming highly elongated coenocytic cells—among the longest cell types in nature. During growth, laticifers branch extensively into Y-, T-, or H-shaped structures that colonize the embryo and subsequently the entire plant body. In mature embryos and seedlings, a dense laticifer plexus is established beneath and around the shoot apical meristem (SAM), from which new laticifer branches originate that grow and, in synchrony with the growth of other tissues, occupy the SAM-derived new organs. Whole-mount histochemical staining in *E. lathyris* has enabled detailed mapping of laticifer distribution throughout the plant, revealing a continuous, branched tubular network resembling animal circulatory systems (Castelblanque et al. 2016). While early reports proposed an intrusive growth mechanism for tissue penetration of laticifer cells (Wilson et al. 1976; Allen and Nessler 1984), this hypothesis remains uncertain due to the lack of dynamic evidence. The identification of the *POOR-IN-LATEX* (*pil*) and *LOT-OF-LATEX* (*lol*) mutants in *E. lathyris* (Castelblanque et al. 2016, 2018) has provided important insights into the genetic regulation of laticifer development and activity. Remarkably, the absence of laticifer cells in the plant during the vegetative growth phase, as revealed in *pil* mutants, does not impair general plant growth or morphology, suggesting that the acquisition of laticifer cells during evolution may have conferred ecological advantages for defense without being essential for plant viability (Castelblanque et al. 2016, 2021). This exemplifies how cell type specialization can represent an adaptive innovation that enhances specific environmental fitness, albeit with potential trade-off effects (Escaray et al. 2024).

To deepen our understanding on the mechanisms controlling laticifers dynamic growth, in this study we have characterized a newly identified *E. lathyris pil13* mutant and, together with previously characterized non-allelic *pil1* and *pil6* lines, performed comparative transcriptomic analyses that allowed to uncover a cohort of genes highly enriched in, if not exclusive of, laticifer cell, which may be useful for the characterization of this type of cell. Among these, we identified 2 HD-Zip I transcription factors, *El_HAT7* and *El_ATHB13*, as key regulators linking BR signaling with cell wall remodeling and laticifer growth. We propose that this HAT7/ATHB13/BR module acts as a central integrator of laticifer growth, coordinating nuclear division and cell wall biosynthesis to sustain the continuous polarized expansion of this coenocytic cell type.

## Results

### Identification of *pil13*, a new mutant defective in laticifer growth

To expand the genetic framework underlying laticifer differentiation, we characterized a newly identified *poor-in-latex* mutant of *E. lathyris*, named *pil13*. Similar to *pil1* and *pil6* mutants (Castelblanque et al. 2016, 2021), *pil13* plants displayed normal vegetative and reproductive growth but completely lacked latex exudation from leaves upon incision (Fig. 1a, b). Backcrossing with wild-type plants and segregation analysis of the F_2_ progeny demonstrated that the *pil13* mutation behaves as a single recessive locus. Among 230 F_2_ plants, 177 displayed a wild-type phenotype and 53 exhibited the *pil* phenotype, fitting a 3:1 segregation ratio (χ^2^ test, *P* < 0.0001). Reciprocal crosses between *pil13* and either *pil1* or *pil6* mutants produced F_1_ plants with wild-type phenotypes, indicating that *pil13* is not allelic to *pil1* or *pil6*. Whole-mount preparations of apical leaves stained with Sudan Black B, which stains specifically laticifer cells accumulating triterpenoids (Castelblanque et al. 2018), revealed that the dense, interconnected network of laticifer cells common to leaves of wild-type plants was absent in *pil13* (Fig. 1c). Quantification of laticifer abundance using the laticifer index (LI)—a measure of laticifer cell density across fixed leaf transects (basal, medial, and apical regions)—confirmed the complete absence of laticifer cells in *pil13* leaves (*LI* = 0) compared with high values recorded in wild-type plants (Fig. 1d). Consistently, the expression of several laticifer-specific marker genes, previously identified by Castelblanque et al. (2016), was abolished in *pil13* plants (Fig. 1e). Remarkably, immunohistochemical analysis revealed that laticifer cell differentiation during early embryogenesis was not affected in *pil13* embryos. Early initiation of laticifers at the heart stage of embryo development (Castelblanque et al. 2016) and their organization during subsequent developmental stages were comparable to those in wild-type embryos (Fig. 1f, 1g). Only a minor reduction in the abundance of laticifer cells was observed in mature *pil13* embryos, as reported also for *pil1* and *pil6* mutants (Castelblanque et al. 2016). In *pil* mutants, therefore, the absence of a laticiferous system in vegetative tissues might be due to a post-embryonary defect. The nature of the signal mediating this post-embryonary growth remains unknown.

**Figure 1:**
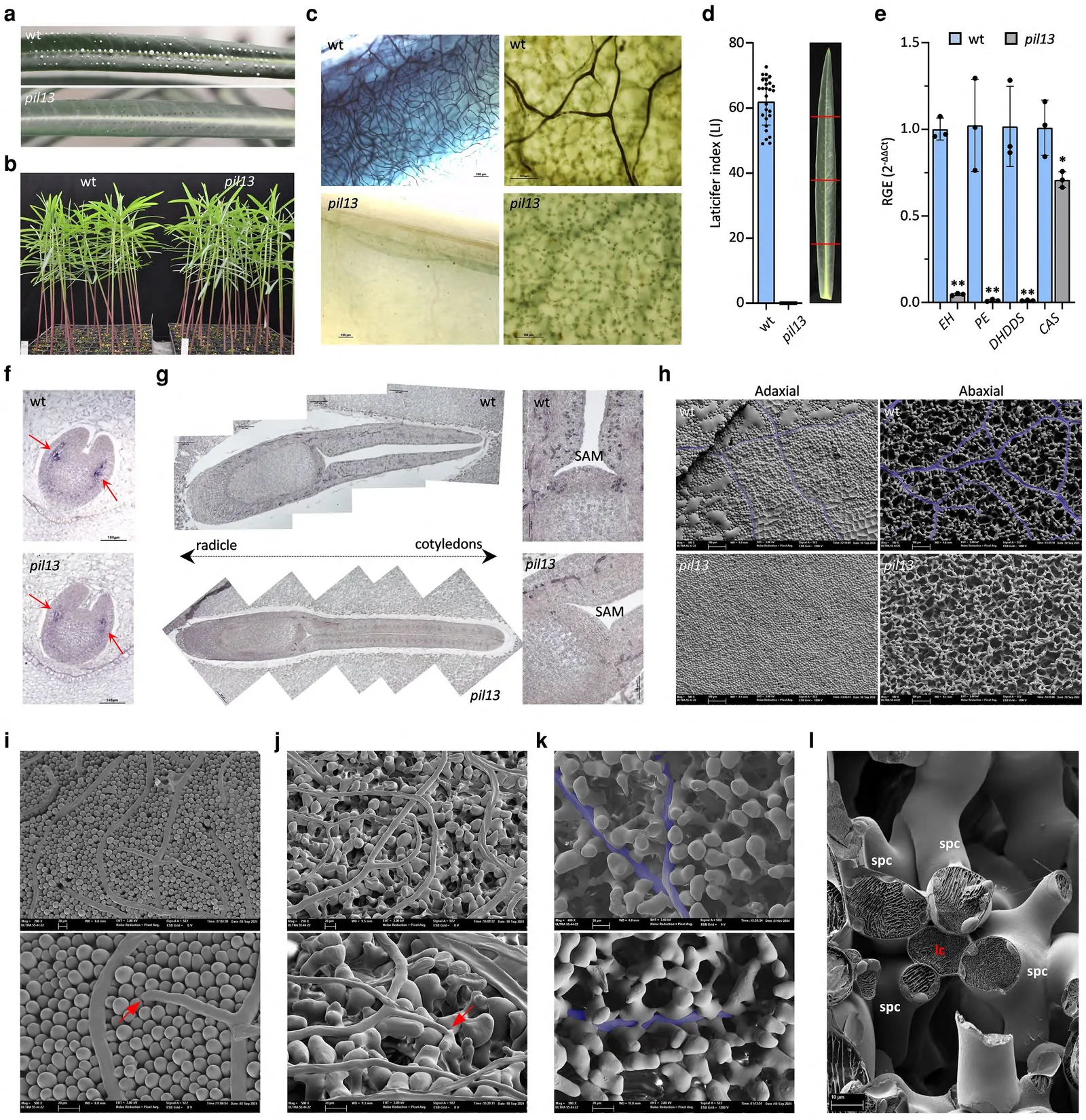
Characterization of the *pil13* mutant and visualization of laticifer cells organization in leaves of wild-type plants. a) Latex oozing from pricked leaves from wild-type plants but not from *pil13* plants. b) Growth and development of wild-type and *pil13* plants at 25 d after germination. c) Whole mount staining with Sudan black B and visualization of the laticifer network organization within developing leaves from wild-type and its absence in *pil13* plants; detail magnification of stained laticifer cells in leaves of each plant is shown on the right. d) Laticifer cell index (LI) determination in leaves of wild-type vs *pil13*. Location of transects along the longitudinal axis of the leaf used to quantify laticifer cells that go across these transects is shown on the right. e) RT-qPCR analysis for previously identified genes from *E. lathyris* associated to laticifer cells (ie *EH*, *PME*, *DHDDS*, and *CAS*). Relative gene expression (RGE) between wild-type plants and *pil13* plants is shown. Bars show the mean ± SEM obtained from 3 biological replicates; asterisks indicate significant differences in the mutant compared with wild type (*P < 0.05, **P < 0.01; pairwise fixed reallocation randomization test). f) Identification of laticifer cell initials (marked with red arrows) differentiating at the early stages of embryogenesis in wild-type and *pil13* developing embryos as revealed by immunohistochemistry using α-LM6 antibodies, which immunodecorate laticifer cells at this developmental stage (Castelblanque et al. 2016). g) Immunolocalization of laticifer cells distributing along the longitudinal axis, from radicle to cotyledons, of wild-type and *pil13* mature embryos. Magnification of images showing laticifer distribution outside the shoot apical meristem (SAM) and along the differentiated cotyledons is shown on the right. Although laticifers are clearly detected in *pil13* embryos, their abundance is not as high as in wild-type embryos. h) Abundance and distribution of superficial laticifer cells, depicted in blue, growing on top of palisade parenchyma tissue and spongy parenchyma tissue as revealed by Cryo-SEM upon peeling the adaxial and abaxial epidermal layer from wild-type and *pil13* leaves. Note the complete absence of laticifer cells in leaves from *pil13* plants. i–k) Hints on the organization and distribution of laticifer cells within leaves: (i) laticifer cells below the upper epidermis in tight contact with palisade parenchyma cells; (j) laticifer cells below the lower epidermis in tight contact and interaction with spongy parenchyma cells. Laticifer cell tips are marked with red arrows; (k) laticifers tubular structures (in blue) distributed internally within the leaf parenchyma tissue are embraced along their longitudinal axis by spongy parenchyma cells following a pattern reminiscent of the bundle sheath surrounding vascular vein. l) Transversal view of an internal laticifer cell (lc) surrounded by 4 of these spongy parenchyma cells (spc), which are rich in organelles and chloroplast.

In leaves of wild-type plants, upon peeling the epidermal layer, cryo-scanning electron microscopy (CryoSEM) allowed direct visualization of laticifers (Fig. S1), which localize immediately beneath both the adaxial and abaxial epidermis and closely associate with palisade and spongy mesophyll cells, respectively (Fig. 1h). The laticifers form a network, which is absent in *pil13* leaf samples, that expands along and across the entire leaf blade (Fig. 1h). The abundance of laticifer cells in the abaxial side appear more prominent than in the adaxial side, which might point toward the existence of a dorsoventral growth effect in the leaf (Fig. 1h). Laticifer cells, although sandwiched between the epidermal cell layer and the parenchyma cells, are with the latter with whom they seem to establish tight cell-to-cell adherences since they remain in the leaf and are not dragged along with the epidermal layer during peeling. Cell contact with neighboring parenchyma cells occurs all along the length of the laticifer cell, including the tips, and also occurs between proximal laticifer cells that grow in parallel or that come across (Figs 1i, 1j). Moreover, superficial laticifers twist and dive into inner parenchymal tissues, where they form abundant cell-to-cell junctions with the internal spongy mesophyll cells (SMCs) (Fig. 1k). These SMCs arrange and embrace the tubular laticifers along their longitudinal axes in a way that resembles a string of pearls in a necklace. This cell-cell organization is similar to the disposition of parenchyma cells of the bundle sheath that surround the vascular veins. SMCs contacting laticifer cells are interconnected with other SMCs through their middle lamella, which in turn serve as bridges to connect other proximal laticifer cells. These SMCs embracing the laticifers are rich in organelles and chloroplasts (Fig. 1l), as is the case of the bundle sheath. We hypothesize that this specialized cellular arrangement might serve not only to anchor laticifer cells through cell-to-cell adhesion but also to conduct fluxes of photosynthates and water into and out of the laticifer cells to maintain their characteristic hydraulic capacity that regulates the internal turgor pressure. These observations, which unveil a highly complex web of cell-to-cell interactions for laticifer cells, suggest that the establishment of the laticifer network must be synchronized with the growth of the other tissues during leaf development.

### Comparative transcriptomic analysis reveals a laticifer-specific gene regulatory network

The availability of *pil* mutants provided an exceptional opportunity to identify core regulators associated with laticifer cell activity and growth. Toward this end, we performed comparative transcriptomic profiling (RNA-seq) of wild type, *pil1*, *pil6*, and *pil13* with RNA extracted from the first and second newly formed leaves. Differential expression analysis revealed sets of upregulated and downregulated genes for each individual *pil* mutant (Fig. 2a). Intersection analysis of these datasets identified a core set of 311 differentially expressed genes (DEGs) common to the 3 *pil* mutants, comprising 285 downregulated and 26 upregulated genes (Fig. 2b, c; Table S1). Validation of the RNA-seq data by RT-qPCR for 25 randomly selected DEGs confirmed consistent expression trends (Fig. S2), providing strong support for the transcriptomic dataset. To determine which DEGs correspond to transcripts preferentially expressed in laticifer cells, we compared transcript abundance (TPM, transcripts per million) for the 285 downregulated genes against RNA-seq data derived from purified latex of wild-type plants, used as a proxy for the laticifer cytoplasm. Nineteen DEGs were absent from latex-derived transcriptomes (Fig. 2d), indicating expression in non-laticifer cells influenced by the presence of laticifers. The remaining 266 DEGs were enriched in latex, and 81 of these exhibited complete transcript absence in *pil13* mutants, identifying them as laticifer-highly specific or, tentatively, laticifer-exclusive genes (Fig. 2d). Comparative analysis among individual *pil* mutants indicate that *pil13* shows the highest repression of laticifer-enriched genes (Fig. 2e), suggesting a stronger phenotype associated to this mutant. In situ hybridization of a representative member of the identified DEGs (ie *Casp31220*) confirmed laticifer-enriched expression in embryos (Fig. S3).

**Figure 2:**
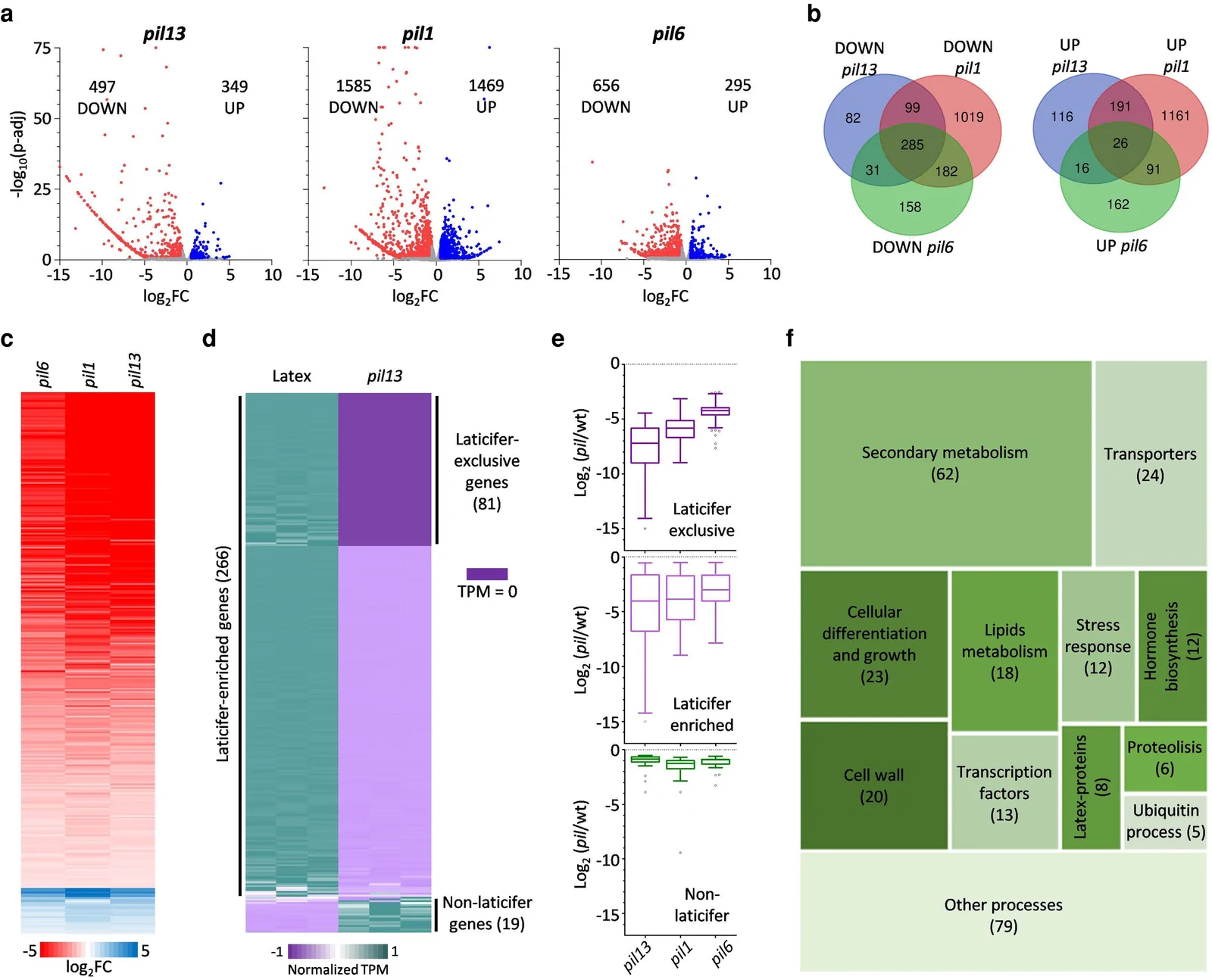
Comparative transcriptomic profiling of wild-type, *pil1*, *pil6*, and *pil13* plants and identification of common DEGs associated to laticifer cells. a) Volcano plots of up- and down-regulated genes in *pil13*, *pil1*, and *pil6* plants compared with wild-type plants. Differentially expressed genes (DEGs) with a *P*-adj value ≤0.2 and a log fold change ≤−0.5 (red point) or ≥0.5 (blue point) are shown. b) Venn diagrams of common up- and downregulated genes in *pil13*, *pil1*, and *pil6* plants. The number of common deregulated genes among the 3 *pil* mutants are indicated in the intersections of the 3 circles. c) Heat map clustering of common up- (blue) and downregulated (red) genes among *pil1*, *pil6*, and *pil13*. d) Heat map cluster analysis reflecting normalized TPM (transcript per million) values derived from latex transcriptome (n = 3) for the 285 genes commonly downderegulated in the 3 *pil* mutants. Further comparison of TPM values recorded in latex with the TPM values recorded in *pil13* plants (n = 3) revealed 81 laticifer-exclusive genes. e) Comparative analysis of log_2_ foldchange corresponding to each group of laticifer-related genes in *pil13*, *pil1*, and *pil6* mutants. f) Grouping of DEGs based on their functional annotation. The number of DEGs genes grouped in each category is indicated within the individual boxes.

Functional annotation of the DEGs revealed that pathways associated with secondary metabolism were among the most significantly overrepresented categories (Fig. 2f; Table S1). This result aligns with the known role of laticifer cells in the synthesis and storage of triterpenoids and other specialized metabolites. Genes associated with lipid metabolism were also prominent, reflecting active membrane biogenesis and vesicle trafficking. In agreement, 4 genes related to autophagy processes were also detected, suggesting dynamic membrane turnover in laticifers. Genes encoding diverse membrane transporters were also overrepresented, significantly enriched for Major Facilitator Superfamily transporters, which mediates solute transport across membranes using electrochemical gradients (Niño-González et al. 2019). These findings indicate that laticifer cells are metabolically active structures highly engaged in nutrient uptake and intercellular transport, which is congruent with the highly interdigitation of laticifer cells with SMCs (Fig. 1j–l). Several genes related to cell differentiation and cell wall remodeling form part of this DEG set (Fig. 2f). In addition, we identified 13 DEGs encoding transcriptional factors (TFs) (Fig. 3a). Among them, *Casp23831* and *Casp02478* displayed the highest TPM values in latex, marking them as strong candidates for laticifer-specific regulators. Orthology analysis identified these 2 TFs as close homologs of AtHAT7 (*Casp23831* namely *El_HAT7*) and ATHB13 (*Casp02478* namely *El_ATHB13*), known BR-responsive HD-Zip I TFs implicated in cortical cell expansion in *Arabidopsis* roots (Nolan et al. 2023). HD-Zip I TFs forms a distinct 12-member clade among the family of HD-Zip TFs in *E. lathyris* (Fig. S4). Among them, only *El_HAT7* and *El_ATHB13* exhibited compromised expression in the 3 *pil* mutants (Fig. 3b). TF-binding site (TFBS) enrichment analysis in the promoters of the 266 laticifer-enriched DEGs using the TDTHub platform, revealed significant enrichment of binding motifs corresponding to 10 TF families based on S-scores (Grau and Franco-Zorrilla 2022) (Fig. 3c), with the highest S-score obtained for binding site recognized by HD-Zip TFs (Table S1). When motif enrichment was evaluated separately for each HD-Zip subclass (I–IV), only the HD-Zip I motifs showed significant enrichment (S-score = 3.40, Fig. 3c). Moreover, restricting the analysis to the 81 DEGs previously classified tentatively as laticifer-exclusive (Fig. 2d) yielded even higher enrichment scores (S-score >6) for binding motifs recognized by *Arabidopsis* HD-Zip I TFs such as *HAT5*, *RCO*, *ATHB51*, *HB34*, and *ATHB13* (Fig. 3d). This led us to consider El_HAT7 and El_ATHB13 as central regulators in laticifer cells.

**Figure 3:**
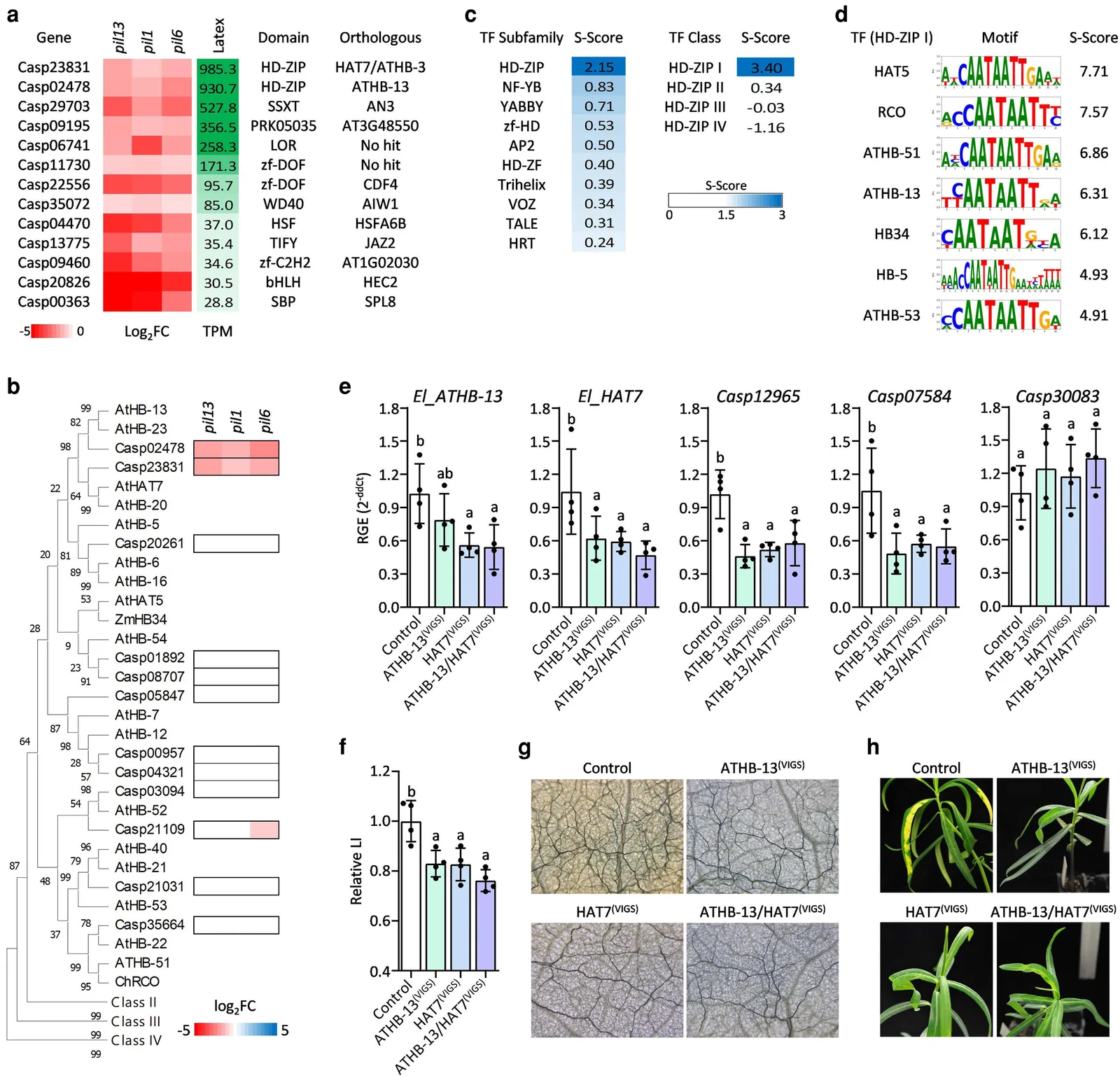
Laticifer-related DEGs encoding transcriptional factors (TFs) and functional implication of *El*_HAT7 and *El*_ATB13 HD-Zip in laticifer growth. a) Gene annotation of the 13 laticifer-associated DEGs encoding TFs and heat map analysis of their comparative repressed expression (log2 FC ≤ −0.5) in *pil13*, *pil1*, and *pil6* plants. TFs were order according to the TPM values recorded in latex, from the highest values to the lowest. The presence of characteristic protein domains defining each TF is indicated along with the orthologous TF from Arabidopsis, which includes: 2 HD-Zip I (HAT7 and ATHB13); ANGUSTIFOLIA3 (AN3), SHOOT GRAVITROPISM 5 (SGR5), HEAT STRESS FACTOR HSFA6b, ABA-INDUCED WD40-REPEAT 1 (AIW1), JASMONATE ZIM-DOMAIN2 (JAZ2), HECATE2 (HEC2*),* ZINC-FINGER OF ARABIDOPSIS THALIANA1 (ZAT9), SQUAMOSA PROMOTER-BINDING-LIKE PROTEIN 8 (SPL8), and 2 DOF zinc finger domain containing TFs homologous to ACDF4. Of these, Casp23831 and Casp02478, encoding HAT7 and ATHB13 HD-Zip I orthologues show the highest expression in laticifer cells. b) Expression analysis (Log_2_FC) of the 12 HD-Zip I class TFs from *E. lathyris* in the 3 *pil* mutants. c) Transcription factor–binding site (TFBS) enrichment analysis using FIMO algorithm in the −1,000 bp of the promoters of the DEGs associated to laticifer cells. Based on S-scores, shown are most significant enriched binding motifs, corresponding to 10 TF families, of which HD-Zip TFs reach highest enrichment. Separate analysis of the 4 subclasses of HD-Zip TFs (I to IV) indicates that only HD-Zip I class TF show significant enrichment (S-score = 3.40). d) Restricting the analysis to the 81 laticifer-exclusive DEGs (Fig. 2d) reveals S-score enrichment >6 for TFBSs recognized by *Arabidopsis* HD-Zip I TFs, including *HAT5*, *RCO*, *ATHB51*, *HB34*, and *ATHB13*. The corresponding HD-Zip I TFBS sequence motifs are illustrated as logos with the height of each base indicative of the probability of the presence of the corresponding base at the designated position. e) Relative gene expression (RGE) of *El_HAT7, El_ATHB13*, *Casp12965*, *Casp07584*, and *Casp30083* upon virus-induced gene silencing (VIGS) in *El_HAT7^(VIGS)^*, *El_ATHB13^(VIGS)^*, *El_HAT7^(VIGS)^*/*El_ATHB13^(VIGS)^*, and control *MgCHL^(VIGS)^* plants. Bars show the means ± SEM obtained from 4 independent plants, different letters indicate significant differences (*P* < 0.05; pairwise fixed reallocation randomization test). f) Determination of laticifer index (LI), relative to control plants, in *El_HAT7^(VIGS)^*, *El_ATHB13^(VIGS)^*, and *El_HAT7^(VIGS)^*/*El_ATHB13^(VIGS)^* plants. Bars show the mean ± SEM obtained from 4 biological replicates; different letters indicate significant differences (*P* < 0.05; ANOVA test, Bonferroni test). g) Sudan black staining of leaves and visualization of laticifer cells from control, *El_HAT7^(VIGS)^*, *El_ATHB13^(VIGS)^*, and *El_HAT7^(VIGS)^*/*El_ATHB13^(VIGS)^* plants. h) Growth of *E. lathyris* VIGS plants 15 d after inoculation to induce gene silencing.

### Silencing of *El_HAT7* and *El_ATHB13* affects laticifer growth

*E. lathyris* is recalcitrant to stable genetic transformation but amenable for virus-induced gene silencing (VIGS) (Escaray et al. 2024). Therefore, we silenced *El_HAT7* and *El_ATHB13*, either individually or simultaneously, coexpressing a visible marker gene encoding the Mg^2+^-chelatase subunit I (*MgCHL*) that produces leaf chlorosis upon repression (Escaray et al. 2024). In *El_HAT7^(VIGS)^* plants (Fig. 3e), where *El_HAT7* transcript accumulation showed a 40% reduction compared with control *MgCHL^(VIGS)^* plants, expression of laticifer marker genes *Casp12965* and *Casp07584* was markedly reduced (55% and 54%, respectively). Interestingly, expression of *El_ATHB13*, which is enriched in laticifers, was also partially repressed in these *El_HAT7^(VIGS)^* plants (Fig. 3e). Conversely, the non-laticifer gene *Casp30083*, which is upregulated in *pil* mutants, showed a similar upregulation pattern in *El_HAT7^(VIGS)^* plants. Likewise, in *El_ATHB13^(VIGS)^* silenced plants, *El_ATHB13* transcript abundance was reduced by 45% relative to controls, a reduction mirrored by decreased expression of *El_HAT7* and the 2 other laticifer marker genes (Fig. 3e). In the combined *El_HAT7/ElATHB13^(VIGS)^* double-silenced plants, expression of both *El_HAT7* and *El-ATHB1*3, as well the selected laticifer and non-laticifer marker genes, reproduced the expression pattern observed in individual silenced plants. Laticifer index (LI) recording in the newly formed leaves of silenced plants (Fig. 3f, g), revealed a significant reduction of 17% in both *El_HAT7^(VIGS)^* and *El_ATHB13^(VIGS)^* plants compared with controls (Fig. 3f). LI reduction was also notable (24%) in *El_HAT7/El_ATHB13^(VIGS)^* double-silenced plants (Fig. 3f). Sudan Black B staining revealed that the remaining laticifer network in the silenced plants still accumulated substantial levels of triterpenoids (Fig. 3g), indicating that triterpenoid metabolism was not notably impaired in silenced plants. With the caveat that gene silencing mediated by VIGS in general is not complete, these experiments suggest that *El_HAT7* and *El_ATHB13* are involved in regulating laticifer growth. The downregulation of both TFs did not produce major developmental abnormalities, except for mild leaf curling in some of the newly formed leaves of *El_HAT7^(VIGS)^*, *El_ATHB13^(VIGS)^*, and *El_HAT7/El_ATHB13^(VIGS)^* plants (Fig. 3h), which was absent in control *MgCHL^(VIGS)^* or *pil13* plants. These results collectively support a role for *El_HAT7* and *El_ATHB13* for laticifer growth.

### Laticifer cell growth is promoted by 24-epibrassinolide (EBL) and inhibited by brassinazole (BRZ)

In Arabidopsis, HAT7 and ATHB13 function within the BR-directed gene regulatory network (Henriksson et al. 2005; Ariel et al. 2007; Ribone et al. 2017; Nolan et al. 2023). To test if *El_HAT7* and *El_ATHB13* are BR-responsive genes, we treated *E. lathyris* with 10 μM 24-epibrassinolide (EBL). *El_HAT7* and *El_ATHB13* transcript levels showed transient increases, peaking at 24 h and declining by 48 h after EBL application (Fig. 4a), confirming that both TFs are BR-responsive factors. This transient activation was mirrored by the laticifer markers (*Casp12965* and *Casp19002*) but not by the non-laticifer marker *Casp30083* that remained unchanged (Fig. 4a). Then, we evaluated whether BRs might influence laticifer growth by treating *E. lathyris* plants with EBL for 15 d (sprayed every 2 d). The first and second pairs of newly formed leaves (nodes 3 and 2, respectively) and the older leaves at node 1 were analyzed for laticifer abundance (Fig. 4b). Newly formed leaves at nodes 3 and 2 from EBL-treated plants exhibited a 75% and 43% LI enhancement, respectively, compared with mock-treated controls (Fig. 4b). In contrast, leaves at node 1, which were already formed at the time of EBL treatment, showed no significant LI change (Fig. 4b). EBL-mediated LI enhancement was also noticed using cryoSEM, which showed a denser network of laticifers (Fig. 4c). Moreover, EBL-mediated LI enhancement was accompanied by a sustained upregulation of *El_HAT7* and *El_ATHB13* (Fig. S5). Leaf expansion was similarly enhanced by EBL (Fig. 4d), suggesting that EBL-mediated stimulation of LI enhancement occurs in coordination with leaf development. In marked contrast to the EBL-mediated enhancement of LI, the epidermal cell index (EI) (Fig. 4e) and palisade mesophyll cell index (PMI) (Fig. 4f) that measure the number of each of these 2 cell types along the leaf width axis, revealed no variation mediated by EBL. However, the epidermal cells showed a 33% increase in cell size area (Fig. 4g), and palisade parenchyma cells also exhibited significant cell size enlargement (21%; Fig. 4h) following EBL treatment. Therefore, EBL-induced leaf growth (Fig. 4d) results from cell expansion enhancement rather than enhanced cell proliferation of epidermal and mesophyll cells. To further assess the dependence of laticifer growth on BR availability, plants were treated with 5 μM brassinazole (BRZ), a BR biosynthesis inhibitor (Asami et al. 2000), by irrigation for 15 d. BRZ-treated plants showed a reduction in leaf size (Fig. 4i), confirming inhibition of BR activity. This reduction was accompanied by a significant 29% decrease in LI value (Fig. 4j), whereas EI and PMI values remained unchanged. Together, these data demonstrate that BR availability strongly influences laticifer growth during leaf development.

**Figure 4:**
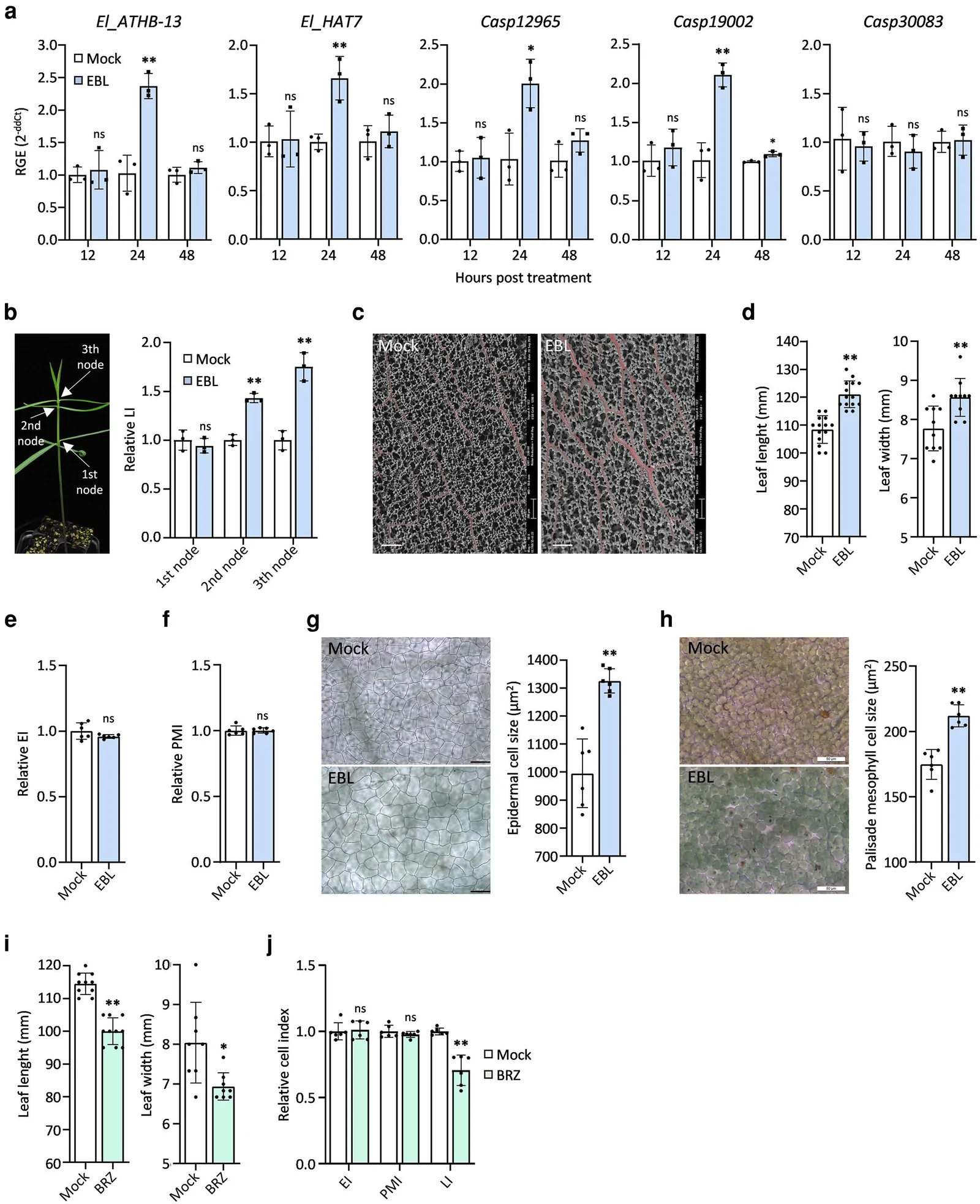
Effect of EBL and BRZ in laticifer cell growth. a) Relative gene expression (RGE) of *El_HAT7*, *El_ATHB13*, *Casp12965*, *Casp19002*, and *Casp30083* in leaves of *E. lathyris* plants at 12, 24, and 48 h after application of 10 μM EBL. Bars show the means ± SEM obtained from 3 biological replicates; asterisk indicate significant differences compared with control (mock) plant (*P < 0.05, **P < 0.01; pairwise fixed reallocation randomization test). b) Laticifer cell index (LI) determination, relative to mock treated plants, at 15 d after application of 10 μM EBL in the newly formed laves corresponding to nodes 3 and 2, as indicated on the picture on the right, and in leaves inserted in node 1 that were already formed at the time of EBL’s first application. Bars show the means ± SEM obtained from 3 biological replicates; asterisk indicate significant differences compared with control (mock) plants (** *P* < 0.01; Student *t* test). c) Cryo-SEM images of the spongy parenchyma tissue as observed after peeling the lower epidermal layer of leaves from control (mock) and EBL-treated plants. The network of laticifer cells is depicted in red. d) Leaf length and width determination in control and EBL-treated plants. Bars show the means ± SEM obtained from 14 (length) and 10 (width) biological replicates; asterisk indicate significant differences compared with control (mock) plants (**P < 0.01; Student *t* test). e, f) Epidermal cell index (EI) (g) and palisade mesophyll cell index (PMI) (h) determination, relative to mock-treated plants, at 15 d after application of 10 μM EBL in newly formed laves corresponding to node 2 (as indicated in b). Bars show the means ± SEM obtained from 6 biological replicates (ns: no significant difference; Student *t* test). g, h) Recording of epidermal (g) and palisade mesophyll (h) cell size in control and EBL-treated plants. Representative photographs of epidermal cell layer and palisade parenchyma are shown on the left of each graph. Bars show the means ± SEM obtained from 6 biological replicates; asterisk indicate significant differences compared with control (mock) plants (**P < 0.01; *t* test with Welch correction for d and Student *t* test for e). i) Effect of BRZ treatment (5 μM), applied by soil irrigation for 15 d, on leaf growth. Bars show the means ± SEM obtained from 10 (length) and 8 (width) biological replicates; asterisk indicate significant differences compared with control (mock) plants (*P < 0.05, **P < 0.01; Student *t* test). j) BRZ treatment reduces the laticifer index (LI) but not on epidermal (EI) or palisade mesophyll cell (PMI) indexes. Bars show the means ± SEM obtained from 6 biological replicates; asterisk indicate significant differences compared with control (mock) plants (**P < 0.01; Student *t* test).

### EBL promotes activation of mitotic marker genes coupled to cell expansion specifically in laticifer cells

Since laticifers are coenocytic cells (Mahlberg and Sabharwal 1966; Castelblanque et al. 2016) that grow by extensive polarized elongation and branching, the substantial EBL-mediated LI enhancement may suggest that for this particular cell type, EBL could differentially be promoting nuclear division coupled to cell expansion. To test this, we quantified nuclear number and the spacing between nuclei along individual longitudinal laticifer cell structures. In leaves from control plants, DAPI staining (Fig. 5a) revealed the oblate-shaped nuclei characteristic of laticifer cells (Castelblanque et al. 2016), which become oriented along the laticifer cell axis and are regularly distributed along the longitudinal laticifer cell with an average 188 ± 40 μm spacing between nuclei. In EBL-treated plants, in which laticifer cells show marked LI enhancement (Fig. 4b), the elongating laticifer cells retained a similar distance between nuclei compared with mock-treated plants (Fig. 5b). This indicates that EBL promotes coordinated nuclear division alongside cell elongation in laticifer cells, contrasting with their effect on epidermal or parenchyma cells, where only cell expansion occurs. Consistently, in wild-type plants at 24 h after application, EBL induced a 41%, 23%, and 36% enhanced expression of the mitotic marker genes *CDT1* (which marks G1/S transition), *CYCB1* (which marks G2/M transition), and *AURORA* (which marks the end of the M phase), respectively (Fig. 5c). This common upregulation of mitotic markers following EBL treatment mirrors the enhanced expression of *El_HAT7* and *El_ATHB13* (Fig. 5c). Interestingly, when EBL was applied to *pil13* plants, which is defective in laticifer cells, no enhancement in *CYCB1*, *CDT1*, and *AURORA* gene expression was observed (Fig. 5c). Neither *El_HAT7* nor *El_ATHB13* showed upregulation following hormone application in the mutant (Fig. 5c). Moreover, basal expression level of the mitotic marker genes was significantly lower in *pil13* control plants compared with wild-type control plants (Fig. 5c). Notably, *pil13* mutant is not defective in BR perception, since mutant plants respond to EBL-mediated leaf expansion enhancements as wild-type plants (Fig. S6). Moreover, expression of *Casp26619*—a non-laticifer BR-induced gene—was similarly enhanced in wild-type and *pil13* plants upon EBL treatment (Fig. 5c). These results indicate that the observed EBL-mediated activation of the mitotic markers genes takes place primarily in laticifer cells, which reinforces the consideration that BR-promoted growth involves both cell expansion and nuclear division specifically in these cells. These observations might give credence to the early proposal suggesting that laticifers may exhibit a semi-autonomous growth program (Mahlberg and Sabharwal 1966, 1967).

**Figure 5:**
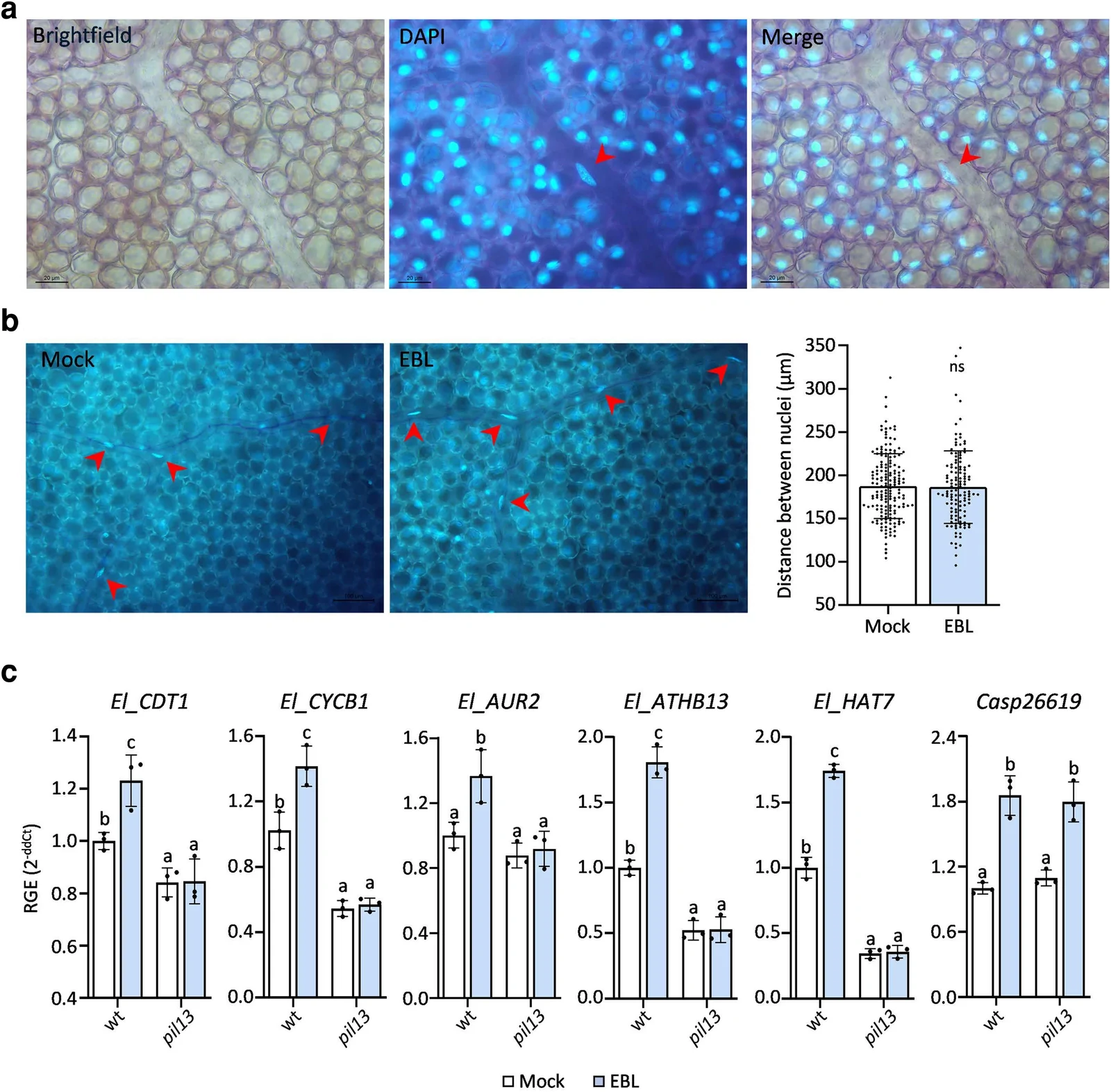
Effect of EBL on laticifer nuclear division. a) DAPI staining of peeled leaves and microscopic identification of nuclei located within longitudinal laticifer cells (indicated by red arrows), showing their characteristic oblate shape. b) DAPI staining and microscopic identification of nuclei within longitudinal laticifer cells (red arrows) in leaves from mock and EBL-treated plants. On the right, the determination of the distance between laticifer nuclei in EBL-treated and mock plants is shown. Bars represent means ± SEM from 150 replicates (ns: no significant difference; Mann-Whitney test). c) Relative gene expression (RGE) of *El_CDT1*, *El_CYCB1*, *El_AUR2*, *El_ATHB13*, *El_HAT7*, and *Casp26619* in leaves of wild-type *E. lathyris* plants and *pil13* mutants 24 h after application of 10 µM EBL. Bars show means ± SEM from 3 biological replicates; asterisks indicate significant differences compared with control (mock) plants (*P < 0.05, **P < 0.01; pairwise fixed reallocation randomization test).

### BRs mediate the expression of genes related to cell wall and pectin biosynthesis in laticifer cells

Plant cell growth relies on the controlled expansion of the cell wall, a process determined by the interplay between turgor pressure and wall extensibility (Ray et al. 1972; Cosgrove 2024). Consistent with the remarkable effect of EBL in promoting cell expansion in laticifers, a cluster of DEGs related to cell wall organization was identified (Fig. 6a), including the following: 2 CELLULOSE SYNTHASES (CESA), in agreement with CESA expression being regulated by HAT7 and BRs (Sun et al. 2010; Xie et al. 2011; Nolan et al. 2023); an ENDO-1,4-β-MANNOSIDASE 7 (MBOAT), which catalyzes the cleavage mannan-type hemicellulose during wall loosening (Rodríguez-Gacio et al. 2012); 2 PECTINESTERASES (PMEs) and 1 PECTINESTERASE INHIBITOR (PMEI), suggesting dynamic de-esterification of homogalacturonan (HG) pectins in laticifers; a β-1,4-GALACTAN SYNTHASE (GalS1) and a RG-I:RHAMNOSYLTRANSFERASE (RRT), which transfers galactose and rhamnose residues onto rhamnogalacturonan-I (RG-I) pectin backbone (Prabhakar et al. 2023), indicates laticifer enrichment in RG-I–type pectin. Similarly, genes encoding polysaccharide acyltransferases and 1 acetyltransferase, homologous to *PROTEIN TRICHOME BIREFRINGENCE* (*TBR*) and *POWDERY MILDEW RESISTANT 5* (*PMR5*)—enzymes involved in pectin acetylation—were also upregulated, highlighting the importance of pectin modification for laticifer wall remodeling. Different glycosyltransferases, 3 PROLINE-RICH PROTEINS (PRPs) carrying a conserved Cys-rich domain (-DC(N9)CN13CC(N5)C(N2)C(N16)C-) reminiscent of PAC domain–containing proteins that interact with pectic polysaccharides during rapid cell expansion (Nguyen-Kim et al. 2020) (Fig. S7), a GLYCINE-RICH PROTEIN (GRP) associated with cell wall function (Fig. S7) and a HYDROXYPROLINE O-ARABINOSYLTRANSFERASE (HPAT), which adds arabinose to cell wall proteins and whose loss causes tip-growth arrest (Sánchez-Rangel et al. 2013), are also part of this laticifer cell wall–related DEG set.

**Figure 6:**
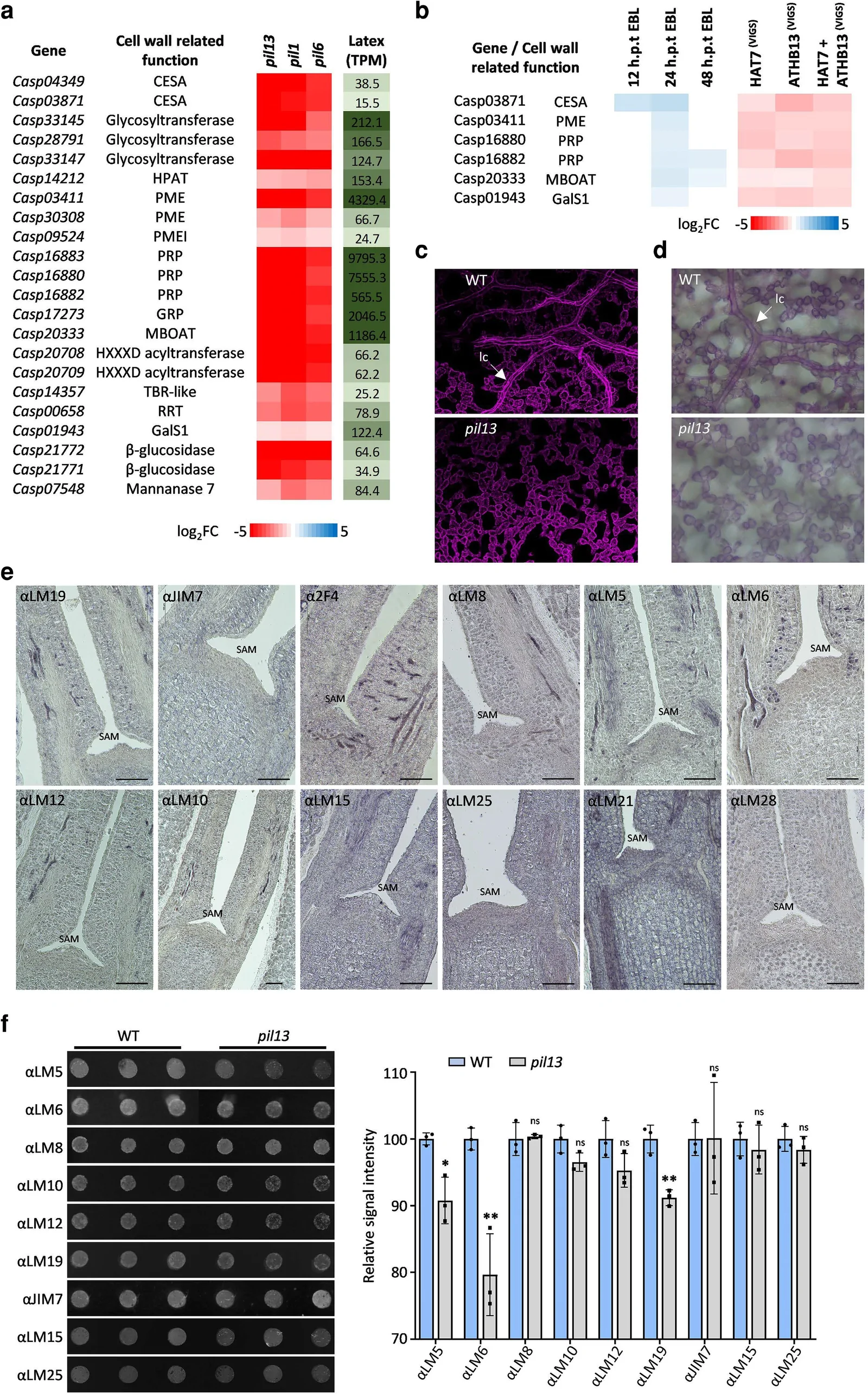
Cell wall–related DEGs and active engagement of laticifer cells in pectin biosynthesis. a) Gene annotation of laticifer-associated DEGs encoding cell wall–related proteins and pectin biosynthesis and remodeling enzymes. Heat map analysis of their comparative repressed expression (Log_2_FC ≤ −0.5) in *pil13*, *pil1*, and *pil6* plants, compared with wild-type plants, along with their TPM values recorded in latex, are shown for comparison. b) RT-qPCR expression analysis for representative members of this laticifer cell wall-DEGs cluster in leaves of wild-type plants at 12, 24, and 48 h after treatment with 10 μM EBL (left panel) and in leaves from *El_HAT7^(VIGS)^*, *El_ATHB13^(VIGS)^*, and *El_HAT7^(VIGS)^*/*El_ATHB13^(VIGS)^* silenced plants (right panel). Each value shows the Log_2_FC obtained from compare with mock-treated plants (n = 3); only significant values (*P* < 0.05; pairwise fixed reallocation randomization test) are shown in blue (upregulated) or in red (downregulated). c, d) Whole-mount pectin-specific staining of wild-type and *pil13* leaves with Coriphosphine O (c) and Ruthenium red (d), showing a remarkable staining of laticifer cells only in leaves from wild-type plants. “lc” denotes laticifer cells. e) Immunohistochemical staining of *E. lathyris* embryos with different antibodies recognizing distinct modifications in HG- and RG-I-type pectins. α-LM6 and α-LM5 strongly immunodecorated laticifer cells. α-LM19, α-LM8, α-LM10, α-LM12, and α-2F4 also labeled laticifer cells in the embryo. Scale bar = 100 µm. f) Dot blot analysis of distinct pectin modifications as present in the cell wall AIR fraction derived from leaves of wild-type and *pil13* plants. Each dot represents 60 μg of a pool AIR fraction derived from 3 independent plants. Nitrocellulose filters were developed with the indicated antibodies (left), and the immunolabeling signal in each dot was optically scanned and analyzed for each of the antibodies. α-LM6 and α-LM5, and α-LM19 rendered a significant signal reduction in *pil13* plants.

Interestingly, expression analysis of some of these laticifer cell wall–related genes revealed transient upregulation following EBL (Fig. 6b). Moreover, their expression was downregulated in *El_HAT7^(VIGS)^*, *El_ATHB13^(VIGS)^*, and *El_HAT7/El_ATHB13^(VIGS)^* plants (Fig. 6b). Together, these results indicate that genes involved in cell wall expansion, remodeling, and pectin metabolism constitute a regulatory module positively controlled by *El_HAT7*/*El_ATHB13* and BRs in laticifer cells.

### Laticifer cell walls are enriched in de-esterified HG- and rhamnogalacturonan-I (RG-I)-type pectins

The cell wall–related transcriptional signature shown above suggests laticifers are actively engaged in pectin biosynthesis, consistent with the high pectin content reported in laticifers located in the apical meristem of *Asclepias speciosa* (Serpe et al. 2002). Pectin enrichment in *E. lathyris* laticifer walls was examined by histochemical staining using the fluorochrome Coriphosphine O (Fig. 6c), which stains pectic components of the primary wall and middle lamella (Ueda and Yoshioka 1976; Weis et al. 1988), and Ruthenium Red (Fig. 6d), which specifically stains non-esterified HGs (Sterling 1970). Both dyes strongly stained laticifer cells in wild-type leaves but not in *pil13* mutant leaves (Fig. 6c, d). The dyes also labeled the middle lamella of spongy parenchyma cells, consistent with the presence of de-esterified pectins in these cells (Jarvis et al. 2003).

Pectins comprise 3 main structural domains—HG, rhamnogalacturonan I (RG-I), and RG-II—with HG and RG-I as the major pectic backbones in Type I primary cell walls (Carpita and Gibeaut 1993; Mohnen 2008). To further characterize pectin composition in laticifers, we performed immunolocalization studies using antibodies directed against distinct pectic epitopes in mature embryos, which are enriched in differentiated laticifer cells surrounded by less specialized cell types. HG, the unbranched homopolymer of α-1,4-linked D-galacturonic acid (GalA) and the most abundant pectic domain (Caffall and Mohnen 2009), was examined with α-JIM7 and α-LM19, which recognize methylesterified and de-methylesterified GalA residues, respectively. Only α-LM19 labeled differentially laticifers (Fig. 6e), indicating de-esterified HG enrichment in their cell walls, which is congruent with highly expressed *PME* genes in laticifer cells (Fig. 6a). α-LM19 also labeled the laticifer in *pil13* embryos, indicating the mutant is not defective in this pectic polysaccharide (Fig. S8). De-esterified HG is more prone to Ca^2+^ cross-linking, potentially conferring local mechanical stability to the cell wall. The presence of de-esterified HG-Ca^2+^ cross-linking was assessed using α-2F4 (Liners et al. 1989), which, like α-LM19, revealed intense immunodecoration of laticifers (Fig. 6e). In addition, α-LM8, which detects xylogalacturonan (a xylose-modified HG involved in cell adhesion; Willats et al. 2006), also recognized laticifer cells (Fig. 6e). Not all laticifer cells were equally decorated by the same antibodies, suggesting that pectin remodeling in laticifers is a dynamic process.

RG-I–type pectin, composed of repeating disaccharides of GalA and rhamnose with side chains of arabinose and/or galactose, was examined with α-LM5 (recognizing (1→4)-β-galactan side chains of RG-I), which labeled both laticifer and provascular cells (Fig. 6e). Moreover, α-LM6, specific for (1→5)-α-arabinan residues linked to rhamnose or galactose within highly branched RG-I, also decorated laticifers (Fig. 6e). As for α-LM19, α-LM6 also labeled laticifer cells in *pil13* embryos (Fig. 1 and Fig. S8). These observations are congruent with high expression of *GalS1* and *RRT* genes—involved in RG-I biosynthesis—in laticifers. RG-I enrichment confers flexibility to plant cell wall (Jones et al. 2003; Verhertbruggen et al. 2009) and its presence in laticifer cell walls might provide elasticity to withstand the high internal turgor pressure derived from latex accumulation. Conversely, α-LM13 and α-LM16, which recognize long linear unbranched arabinan chains in RG-I, respectively (Verhertbruggen et al. 2009), did not label laticifer cells (Fig. S9). Therefore, the contrasting labeling with α-LM6 versus α-LM13 and α-LM16 indicates that RG-I pectin in laticifers is enriched in branched arabinan structures. Notably, α-LM12, which binds to feruloylated RG-I, also labeled laticifer cells (Fig. 6e). Since feruloyl groups mediate covalent cross-linking among wall polymers and contribute to wall strengthening during expansion (Lindsay and Fry 2008), this result might suggest that feruloylation could be acting to protect laticifer cell wall integrity either during growth or during the filling/emptying dynamics of laticifer cells. Immunolabeling of different hemicellulosic xyloglucans motifs with α-LM15, α-LM25, and α-LM21 revealed no laticifer-specific immunostaining (Fig. 6e). Neither did α-LM28, recognizing heteroxylan enriched in glucuronoxylan, label laticifer cells (Fig. 6e). We could only observe laticifer-specific immunodecoration when using α-LM10 or α-LM12, which recognizes (1–4)-ß-D-xylan and feruloylated-xylan, respectively (Fig. 6e), pointing toward enrichment of this type of hemicellulose in laticifer cell walls.

To corroborate these findings, pectin composition was further analyzed in leaves by immunoblotting using alcohol-insoluble residue (AIR) fractions derived from wild-type and *pil13* plants. α-LM19, α-LM5, and α-LM6 labeling revealed a strong signal in wild-type samples, which was significantly reduced in *pil13* samples (Fig. 6f), suggesting that a substantial portion of de-methylesterified HG and RG-I modified with galactan–arabinan side chains in leaves of wild-type plants originates from laticifer cells. Other epitopes, including those detected by α-LM10 and α-LM12, also tended to decrease in *pil13*, though differences were not statistically significant, possibly reflecting broader distribution of these modifications among leaf cell types. Alternatively, we cannot disregard that the specific RG-I modifications observed in laticifer cells of the embryo suffer changes in vegetative tissues. No differences were detected for epitopes recognized by α-LM25, α-LM15, or α-JIM7, consistent with their lack of labeling in laticifers in the embryo. Collectively, these data indicate that laticifer cell walls are enriched in de-methylesterified HG and RG-I pectins carrying highly branched galactan–arabinan side chains.

### Differential distribution of HG- and RG-I–type pectins in laticifer cell walls

To further confirm deposition of de-methylated HG and RG-I polymers in laticifers established in the leaf and ascertain their distribution pattern in their walls, we performed whole-mount immunolabeling in peeled *E. lathyris* leaves. Results shown in Fig. 7a and Fig. S10A revealed a highly specific distribution pattern of de-methylated HG within the laticifer cell walls, as revealed by immunofluorescence microscopy using α-LM19. Laticifers exhibited a peculiar deposition of de-esterified HG, which is present all along the length of the laticifer. The de-esterified HG polymer assembles into a series of discrete contacting ovals of different areas, which appear flattened along the longitudinal cell axis, including the junctions where laticifers bifurcate. Interestingly, this distribution pattern was mirrored by the binding of COS^488^ (Fig. 7b and Fig. S10b), a fluorescent probe that binds highly de-esterified HG (Mravec et al. 2014), which penetrates more deeply in the tissues than antibodies and therefore allows visualization of the distribution of acidic pectin in the entire laticifer cell body. Interestingly, these irregular flattened ovals, peripherally demarcated by the presence of de-esterified HG, are internally occupied by esterified HG as revealed by α-JIM7 immunolabeling (Fig. 7c and Fig. S10c). The esterified HG showed a complementary non-overlapping deposition pattern, accumulating homogeneously within the interstitial gaps define by the de-esterified HG lattice. Moreover, deposition of RG-I, revealed by α-LM6 (Fig. 7d and Fig. S10d), appears to mirror the deposition of esterified HG, also accumulating internally to the ovals demarcated by de-esterified HG. In fact, dual immunolabeling with α-2F4 and α-JIM7 (Fig. 7e) indicates that the ovals boundaries are rich in de-esterified HG-Ca^2+^ cross-linking, therefore conferring local mechanical stability at the edges of the ovals. A Manders coefficient of 0.312 ± 0.003 indicated low spatial overlap between these 2 domains, suggesting a highly regulated modular assembly (confocal z-stack images are shown in Supplemental Dataset S2). Such polymer organization was consistent across both the shank and the tip of the laticifers (Fig. 7f, g). However, the de-esterified HG-enriched ovals conforming the lattice, which are also decorated upon staining with ruthenium red (Fig. 7e and Fig. S10f), appeared smaller toward the tip. We hypothesize that this bipartite organization might likely serve as a mechanical containment system tailored to the unique physiology of laticifers. The rigid, demethylated-pectate lattice potentially acts as a corset or load-bearing reinforcement to withstand the high internal turgor pressure generated by latex accumulation. Meanwhile, the internal domain, enriched in methylated-HG and RG-I, may provide the necessary elastic buffering required to maintain wall integrity during cell expansion or pressure fluctuations. In both their discrete organization and mechanical properties, these pectin components evoke the structure of a compressible mesh ball.

**Figure 7:**
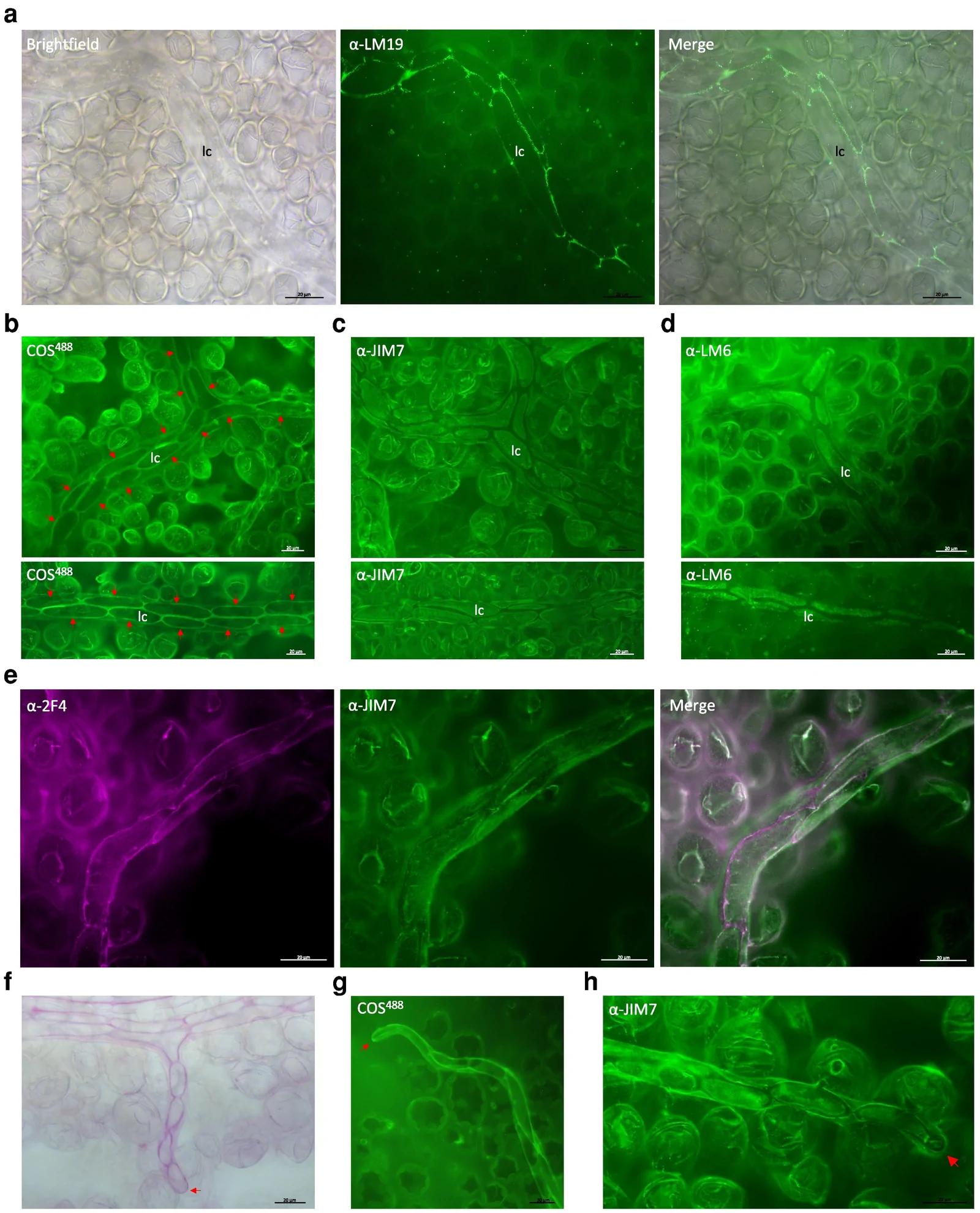
Deposition of pectin polymers in laticifer cells in *E. lathyris* leaves. a) Localization of de-methylated HG accumulation using α-LM19. Left: bright-field microscopy showing a laticifer cells (lc) embedded within the cortical tissue; middle: fluorescence microscopy using α-LM19, revealing a characteristic deposition of de-methylated HG along the laticifer cell wall; right: merging of images. b) COS^488^ labeling of de-methylated HG in a bifurcating mature laticifer cell (up) and a longitudinal laticifer (down) surrounded by parenchyma cells. Red arrows indicate the discrete contacting ovals of de-esterified HG polymers c) Localization of methylated HG accumulation using α-JIM7 in a mature bifurcating laticifer cell (up) and a longitudinal laticifer (down) surrounded by parenchyma cells. d) Localization of RG-I accumulation using α-LM6 in a mature bifurcating laticifer cell (up) and a longitudinal laticifer (down) surrounded by parenchyma cells. e) Double immunolabeling of de-methylated HG crosslinked to Ca^2+^ and methylated HG using α-2F4 and α-JIM7, respectively. Left: DyLight594 fluorescence associated to secondary antibody recognizing α-2F4 showing a laticifer cells (lc) embedded within the cortical tissue; middle: FITC fluorescence associated to secondary antibody recognizing α-JIM7; right: merging of images. f) Ruthenium red staining of an emerging young lateral laticifer. Note the interconnected rings of de-methylated HG distributed along the laticifer cell wall, including the cell tip (red arrow) of the emerging lateral. g) Elongating laticifer labeled with COS^488^ revealing the de-methylated HG connecting rings that are present even close to the cel tip (marked with red arrow). h) Elongating laticifer labeled with α-JIM7 detecting ring-contained methylated HG deposition at the proximity of the cell tip (red arrow). The described pectin deposition and ring-like patterns were consistently present in all the examined laticifer cells (n ≥ 40) derived from at least 4 independent plants.

## Discussion

The differentiation of laticifer cells exemplifies an exceptional case of plant cell specialization, where a highly polarized growth must be sustained over extraordinary durations and distances, all while being surrounded by many other cell types with different fates. During growth, laticifer cells establish abundant cell-to-cell contacts with neighboring cells. High-resolution CryoSEM analysis provides definitive visual evidence of this complex tissue architecture, revealing the intricate distribution and numerous cell-to-cell interactions of laticifer branches as they navigate the intercellular spaces of the mesophyll.

Unlike traditional tip-growing models like pollen tubes or root hairs, which exhibit relatively short and localized growth phases, laticifers must maintain an active growth strategy of tissue occupancy throughout the plant's entire life cycle, in synchrony with SAM activity and coordinated with the growth of the new organs that are continuously derived from it. Moreover, in contrast to parenchymatous cells, laticifers undergo extensive elongation and branching supported by a coenocytic organization. Therefore, laticifer cell fate must be tightly regulated by hormonal cues and transcriptional programs. The finding that laticifer initiation and organization are normal in *pil13* embryos, as reported also for the nonallelic *pil1* and *pil6* mutants (Castelblanque et al. 2016), demonstrates that the genetic program for cell identity specification is decoupled from the program for vegetative expansion. This suggests that while a cell may be correctly specified in the embryo, its ability to develop through growing organs depends on a dedicated growth-control module activated after germination, the absence of which in *pil* mutants leads to the collapse of the laticiferous system without altering basic cellular identity.

The comparative transcriptomic profiling of *pil* mutants revealed a core set of 266 laticifer-enriched genes, a transcriptomic signature that provides an entry point to identify the molecular mechanisms controlling laticifer growth and activity. The prominence of genes associated with lipid and secondary metabolism, along with those related to cell wall remodeling, aligns with the laticifer's role in synthesizing triterpenoids but also reflects the immense demand for active membrane biogenesis, vesicle trafficking, and cell wall biosynthesis required to sustain laticifer growth. From the laticifer-associated transcriptomic signature we identified *El_HAT7* and *El_ATHB13*, encoding 2 distinct HD-Zip I TFs, as essential positive regulators of laticifer growth, since their downregulation using VIGS results in a significant reduction of the laticifer cell index (LI) without affecting the basic metabolic identity of the surviving cells. HD-Zip I TFs are pivotal regulators of cell differentiation programs that respond to environmental and hormonal cues (Henriksson et al. 2005; Ariel et al. 2007) and directly modulate cell wall extensibility through transcriptional control of wall-related enzymes (Olsson et al. 2004). Their orthologs in *Arabidopsis* have been implicated in cell expansion through a BR-dependent pathway (Nolan et al. 2023), suggesting conservation of a mechanistic module that couples hormonal cues with cell wall remodeling. Interestingly, since HD-Zip I TFs also contribute to the regulation of turgor-related genes (González et al. 2019), it is enticing to hypothesize that *El_*HAT7 and *El*_*ATHB13* could provide a transcriptional link between wall remodeling and turgor pressure control, collectively orchestrating the growth of the laticifer system.

BRs regulate cell expansion and differentiation, acting through a network of transcription factors, including HAT7 and ATHB13 (Clouse 2011; Nolan et al. 2023; Wang et al. 2023). In *E. lathyris*, the responsiveness of *El_*HAT7 and *El*_*ATHB13* to 24-epibrassinolide (EBL) confirms that they are integral components of the BR signaling pathway. Moreover, the sensitivity of laticifers to BRs seems to be a central pillar of their growth strategy. Notably, EBL application exerts a significant 75% enhancement of the laticifer cell index (LI) in newly formed leaves, while the epidermal cell index (EI) and the palisade mesophyll cell index (PMI) remain invariant. Conversely, inhibition of BR biosynthesis by BRZ promotes a significant LI reduction. These observations suggest that laticifers possess a BR responsiveness program that might allow them to synchronize their growth with global leaf expansion. Crucially, since laticifers are coenocytic, our data show that BRs differentially promote nuclear division coupled to cell expansion in laticifer cells. The upregulation of mitotic marker genes like *CDT1*, *CYCB1*, and *AURORA* by EBL, and the concurring upregulation of *El_HAT7* and *El_ATHB13* specifically in a laticifer-dependent manner, suggest that BRs, HAT7A, and ATHB13 take part in the overall coordination of the coenocytic program, ensuring that karyokinesis keeps pace with the massive anisotropic expansion of the laticifer cell. This might favor the interpretation that the laticiferous system behaves as a growing unit that responds to hormonal stimuli differently than other tissues, which reconciles with the early proposal by Mahlberg and Sabharwal (1966, 1967) that laticifers may exhibit a semi-autonomous growth program.

The mechanical foundation of this laticifer cell expansive growth, along with its ability to support the elevated turgor pressure required for its functional role in defense, might be rooted in a unique cell wall composition. Unlike other tip-growing cells, laticifer cell walls exhibit a peculiar spatial segregation of pectic polysaccharides enriched in de-esterified HG and RG-I polymers. The observed deposition of these 2 polysaccharides, following a complementary, non-overlapping pattern, confers a genuine modular architecture to laticifer cell walls. The linear de-esterified HG polymer assembles into a series of discrete contacting ovals that might function as fastening devices to provide the necessary hoop stress resistance and prevent radial failure. On the other hand, the highly branched RG-I, along with esterified HG, which act as a flexible, cohesive matrix, are encased within the HG ovals, suggesting this organization might constitute the internal expansive matrix that accommodates internal changes in turgor pressure, allowing the laticifer to inflate without losing its structural axis. Furthermore, this transverse pectic cell-wall reinforcements that form an oval-based architecture might provide additional advantages as it seems to be compatible with the high flexibility and mechanical sinuosity of laticifers, allowing the cell to execute sharp turns (twists) to navigate obstacles as they grow internally surrounded by other tissues. Furthermore, the identification, among the laticifer DEGs, of a series of genes configuring a cell wall–related transcriptional signature, including PMEs and GalS1, which appear to be regulated by the HAT7/ATHB13/BR module, indicates that laticifers employ a sophisticated enzymatic toolkit for their cell wall construction and remodeling.

Taken together, our findings suggest a cellular model where hormonal signaling, transcriptional reprogramming and cell wall remodeling are mechanistically coupled. HAT7/ATHB13 regulate the expression of wall-modifying enzymes to define the mechanical status of the cell wall. In turn, the mechanical state of the pectic matrix likely provides feedback—perhaps through wall-associated kinases or other sensors—that further modulates BR perception and signaling, ensuring that coenocytic growth occurs only when the cell is structurally capable of supporting it. This integrated circuit explains how laticifers can achieve such extreme expansion without structural failure, traversing the plant body while maintaining their coenocytic organization and defensive functionality. From an evolutionary perspective, the recruitment of this HD-Zip I–BR module may represent a key adaptation that allowed to develop a plant-wide defensive network that grows in lockstep with the host, ensuring that no new tissue remains unprotected by the toxic latex. Such a module highlights the remarkable plasticity of plant growth programs and identifies potential genetic targets for modulating laticifer-specific development.

## Material and methods

### Plant material and growth conditions

*E. lathyris pil13* mutant was obtained as described previously for *pil1* and *pil6* mutants (Castelblanque et al. 2016). Wild-type and the 3 *pil* mutant plants were cultivated in a growth chamber (19 to 23 °C temperature, 85% relative humidity, 120 to 150 μmol m^−2^ s^−1^ fluorescent illumination, 16-h light photoperiod). One-month-old *E. lathyris* plants were used for RNA isolation and transcriptomic analysis.

### Leaf staining and laticifer index determination

Laticifers cells were stained with Sudan Black B as described in Castelblanque et al. (2016). After whole-mount staining of leaves, a Leica DM5000 microscope was used for brightfield images. To estimate the laticifer density, 3 transects perpendicular to the leaf lamina were marked (at the base, in the middle, and at the apex; see Fig. 1d). The number of laticifer cells that cross transects were counted under the microscope, at a magnification of 40X, the mean between the 3 transects was considered to calculate the laticifer index (LI).

### Genetic analysis

Segregation of phenotypes in the F2 generation upon backcrossing of *pil13* with the parental line, was analyzed with the *X*^2^ test for goodness of fit. For complementation analysis, *pil* mutants had crossed each other and mutant *pil* phenotypes scored as latex oozing after priking leaves of F1 plants

### Immunohistochemical identification of laticifers in *E. lathyris* embryos

Embryos were prepared for immunohistochemical analysis as previously described (Castelblanque et al. 2016). Basically, embryos were fixed overnight with FAE (formaldehyde:acetic acid:ethanol:water, 3.5:5:50:41.5) and embedded in paraffin. Embryos were sectioned on a HM325 microtome at 7 µm. After paraffin removal, sections were blocked in PBS containing 0.1% (w/v) BSA/0.05% (v/v) Tween 20 and incubated with the monoclonal antibodies in the same solution. An anti-rat IgG conjugated with alkaline phosphatase (Sigma), diluted 1:2000, was used as a secondary antibody, and sections were revealed with nitro-blue tetrazolium and 5-bromo-4-chloro-3-indolyl-phosphate. Specifically for the monoclonal α-2F4, the tissue sections were incubated in the same PBS buffer supplemented with 5 mM Ca_2_Cl and the secondary antibody employed was an anti-mouse IgG conjugated with alkaline phosphatase.

### Whole-mount immunohistochemical analysis of laticifer cells

*E. lathyris* leaves were mechanically peeled to remove the epidermal layer and fixed in FAE as described above. Leaf samples were treated with ethanol to get rid of pigments, then progressively hydrated and finally blocked in PBS containing 5% (w/v) BSA. Leaf tissues were subsequently incubated with monoclonal antibodies, diluted 1:20 in PBS containing 0.1% (w/v) BSA. As a secondary antibody anti-rat IgG (1:100) conjugated with FITC was used and fluorescence detected on a Leica DM5000 microscope using GFP filter upon blue light excitation. Specifically for the monoclonal α-2F4, the leaf tissues were incubated in the same PBS buffer supplemented with 5 mM Ca_2_Cl and the secondary antibody employed was an anti-mouse IgG (1:100) conjugated with DyLight594. For immunolabeling, at least 4 independent biological replicates (plants) were analyzed, with 10 representative laticifers images taken for each plant (n ≥ 40 total laticifers). Images were acquired using a Leica DM5000 microscope at a resolution of 2,720 × 2,048 pixels.

Confocal z-stack images were acquired using a Leica Stellaris 8 FALCON confocal microscope (Leica Microsystems, Germany) at excitation wavelengths of 499 nm (FITC) and 590 nm (DyLight594). Four fields of view from each sample (n = 3 independent biological replicates) were analyzed to calculate the Manders' colocalization coefficient. For image processing, maximum-intensity z-projections were generated using FIJI/ImageJ software. Laticifer cells were manually outlined to define the regions of interest (ROIs). Background subtraction and Costes' automatic thresholding were applied prior to coefficient calculation using the JACoP plugin.

### Cryo-scanning electron microscopy (CryoSEM)

From 1-mo-old *E. lathyris* plants, fully developed leaves from the second node (Fig. 4b) were detached and quickly peeled; immediately, a section around 5 × 5 mm without epidermis was fixed and frozen in liquid N_2_ at −210 °C. Frozen samples were processed in a Cryo Preparation system (PP3010T, Quorum Thechnologies, UK), the surface ice was sublimated at −90 °C in vacuum (1 kPA) during 15 min, and a platinum coating was applied (45 s). After that, samples were transferred to a scanning electron microscope (Zeiss ULTRA 55, Germany) and images were recorded at 3 kV.

### *E. lathyris* transcriptome and RT-qPCR gene expression analyses

For transcriptome analysis, the first 2 pair of newly forming leaves close to the apex from wt, *pil1*, *pil6*, and *pil13* plants (n = 3) were collected and RNA extracted as described by Castelblanque et al. (2018). cDNAs were prepared as described in the Illumina TruSeq RNA Sample Preparation Guide. High-Throughput Sequencing was carried out at Central Service for Experimental Research (SCSIE, University of Valencia, Spain), using NextSeq 550 (Illumina) technology. Adapter sequences and low-quality reads were removed using Cutadapt v4.3 (maxN = 0.1; q5 = 20; q3 = 20; minsize = 20) (Martin 2011) and analyzed for quality control using default parameters of FASTQC v0.12.1 tool (https://www.bioinformatics.babraham.ac.uk/projects/fastqc/). *E. lathyris* genome (Wang et al. 2021) was used as reference for mapping the reads by Spliced Transcripts Alignment to a Reference (STAR v2.7.10b; default parameters) software (Dobin et al. 2013). Differential expression analysis between *pil* mutants and wild-type genotypes was conducted using a default parameter of DESeq2 v1.42.0 (Love et al. 2014). Differentially expressed genes (DEGs) were obtained by applying both *P*-adjusted value ≤0.2 and FC ≥ |0.5| filters. Latex raw data were downloaded from National Center for Biotechnology Information (NCBI; https://www.ncbi.nlm.nih.gov/; BioProject PRJNA450173, and processed as above. Functional annotation was downloaded from the Euphorbiaceae Database Portal (Liu et al. 2024).

Real-time PCR was performed using an ABI 7500 Fast Real-Time Sequence Detection System and TB Green Premix Ex Taq (Takara Bio Inc.). Analysis of relative gene expression was performed according to Pfaffl et al. (2002), using ubiquitin (*UBQ*) and elongation factor (*EFACT*) as housekeeping genes. Primer sequences used are detailed in Table S2. Statistical analysis was performed using the Relative Expression Software Tool (REST) according to Pfaffl et al. (2002).

### TFBS enrichment analysis

From 285 downregulated genes common to the 3 *pil* mutants, the 266 DEGs enriched in latex (Fig. 2d) were selected for TFBSs analysis by the TDTHub (Grau and Franco-Zorrilla 2022). Search was performed by FIMO mapping algorithm in 1-kb promoter region and the enrichment score (S-Score) was used as the preferred statistic. S-Score threshold at 5% in this species corresponds to 3.3.

### Virus-induced gene silencing (VIGS)

VIGS experiments were performed as in Escaray et al. (2024). Briefly, 300-pb fragments for *El_HAT7* and *El_ATHB13* were PCR amplified with oligonucleotides indicated in Table S3 and cloned into the pLX-TRV2 vector. Plasmids were then used to electroporate *Agrobacterium* C58C1 previously transformed with the pLX-TRV1 plasmid. Transformants were used for plant inoculation and *Agrobacterium* C58C1 carrying the pLX-TRV1 and MgCHL + pLX-TRV2 vectors was used as control. Inoculated *E. lathyris* plants were cultured for 2 wk after visualization of chlorosis derived from *MgCHL* silencing. Samples taken from the VIGS plants (n = 4) were immediately frozen in liquid N_2_ for RNA isolation. Visualization of laticifers and LI determination was carried out as described above (n = 4).

### EBL and BRZ treatments

For EBL treatment, a water solution containing 0.02% of SILWET L-77 and 10 μM of 24-Epibrassinoide (EBL) was used to spray 2-wk-old *E. lathyris* plants, a similar solution without EBL was used as mock treatment. To evaluate the gene expression at 12, 24, and 48 h post treatment, only 1 treatment was applied. To evaluate other parameters like leaf expansion, laticifer index, cell size and nuclei distance the treatment lasted for 15 d, plants were sprayed every 2 d with a new dose of 10 μM of EBL or without EBL for mock plants.

Brassinazole (BRZ) treatment was carried out by irrigation of 2-wk-old *E. lathyris* plants with a nutrient solution supplemented with 5 μM of BRZ, control plants were irrigated with a similar solution without BRZ. The treatment was applied for 15 d, watering according to the needs of the plants.

### Cell measurement

Leaf samples used to estimate the laticifer index were also used to take epidermal and palisade mesophyll pictures using a Leica DM5000 microscope (Fig. 4g and 4h). Eight randomly selected pictures of a 400 × 400 μm (160,000 μm^2^) area per leaf sample taken from independent plants (n = 6) were used to count the adaxial epidermal cells and estimate the cell size and epidermal cell index (EI). For the palisade mesophyll cells, the same number of pictures and repetitions were analyzed, but the size was 200 × 200 μm (40,000 μm^2^). The palisade mesophyll cells were counted to estimate the cell size and palisade mesophyll index (PMI). Cell size was determined using ImageJ software.

### RNA in situ hybridization

Digoxigenin-labeled sense and antisense probes generated from a 192-bp fragment of *Casp31220* (amplified by PCR and cloned in pGEM-T Easy vector (Promega)) was used for RNA in situ hybridization as described by Ferrándiz et al. (2000) on 7-µm sections of fixed *E. lathyris* mature embryos. Primers used are described in Table S4.

### Coriphosphine and ruthenium stainining

Epidermal peeled leaf samples from *E. lathyris* plants were immersed in FAE (formaldehyde:acetic acid:ethanol:water, 3.5:5:50:41.5) overnight at 4 °C. Samples were washed with 70% ethanol, hydrated and stained for 2 min with aqueous solution of Coriphosphine O (CAS 5409-37-0) at 0.05%. After that, samples were rinsed with distilled water and mounted on slides to obtain confocal images using a Stellaris 8 FALCON confocal microscope (Leica, Germany), captured at 405-nm excitation wavelengths and a 430- to 530-nm range detection. For ruthenium red stain, peeled leaf samples were stained for 30 min with aqueous solution of Ruthenium red at 0.05%, rinsed with water, and mounted on slides. A Leica DM5000 microscope was used for brightfield images.

### Internuclear distance determination

Ruthenium read stained leaf samples, demarcating laticifer cells, were stained with DAPI (1 µg/mL in 1 M TRIS-HCl pH 8) for 5 min, rinsed with water and mounted on glass slides and visualized under a Leica DM5000 microscope using UV. The length of each laticifer and the associated nuclei was measured using the Image J software. The distance between nuclei was estimated as laticifer length/number of nuclei.

### Preparation of AIR and dot blot experiments

Leaves from 1-mo-old *E. lathyris* wild-type and *pil13* mutant were ground in liquid N_2_ and a 100-mg aliquot from each sample was used for alcohol insoluble residue (AIR) extraction as described by Foster et al. (2010) . For dot blot analysis, the AIR fraction was diluted in TRIS-HCl, pH 8, to a final concentration of 600 µg/mL, and an aliquot of 100 µL per sample was spotted onto a nitrocellulose membrane. After drying overnight, the membrane was blocked with 5% milk (w/v) and 0.05% of Tween-20 (w/v) in 1× TBS, and incubated with αLM5, αLM6, αLM8, αLM10, αLM12, αLM19, αJIM7, αLM15, and αLM25 antibodies (diluted 1:100 in 2% milk (w/v) and 0.05% of Tween-20 (w/v) in 1× TBS). After 3 × 5 min wash (0.05% of Tween-20 (w/v) in 1× TBS), the membrane was incubated with a goat anti-Rat IgG—alkaline phosphatase conjugated antibody (diluted 1:10,000 in 2% milk (w/v) and 0.05% of Tween-20 (w/v) in 1× TBS). Signal intensity was measured in photographed nitrocellulose membranes using the integrated density function of Image J software.

### Statistical analysis

Homogeneity of variance was evaluated using a Levene test (Levene 1960) and normal distribution was evaluated using the Shapiro-Wilks test (Shapiro and Wilk 1965). The number of biological repetitions evaluated and particular statistical parameters are reported in the figures and their legends. Each experiment was performed at least 2 times.

## Supplementary Material

koag289_Supplementary_Data

## Data Availability

The raw sequencing data of *E. lathyris* transcriptome analysis have been deposited in the National Center for Biotechnology Information (NCBI; https://www.ncbi.nlm.nih.gov/). The Sequence Read Archive (SRA) files can be downloaded from the BioProject PRJNA1363096.
