# Supplementary material for: A HAT7/ATHB13/brassinosteroid signaling module mediates growth of laticifer cells in Euphorbia lathyris
Source: Plant Cell. 2026 Oct 17;38(10):koag289. doi: 10.1093/plcell/koag289 (PMC13711675; doi:10.1093/plcell/koag289)
Supplement: Supplementary file 1 [file koag289_supplementary_data.zip › Supplemental material 1 - 01-09-2026.pdf]

SUPLEMENTARY MATERIAL

SUPPLEMENTAL FIGURES 1 to 9

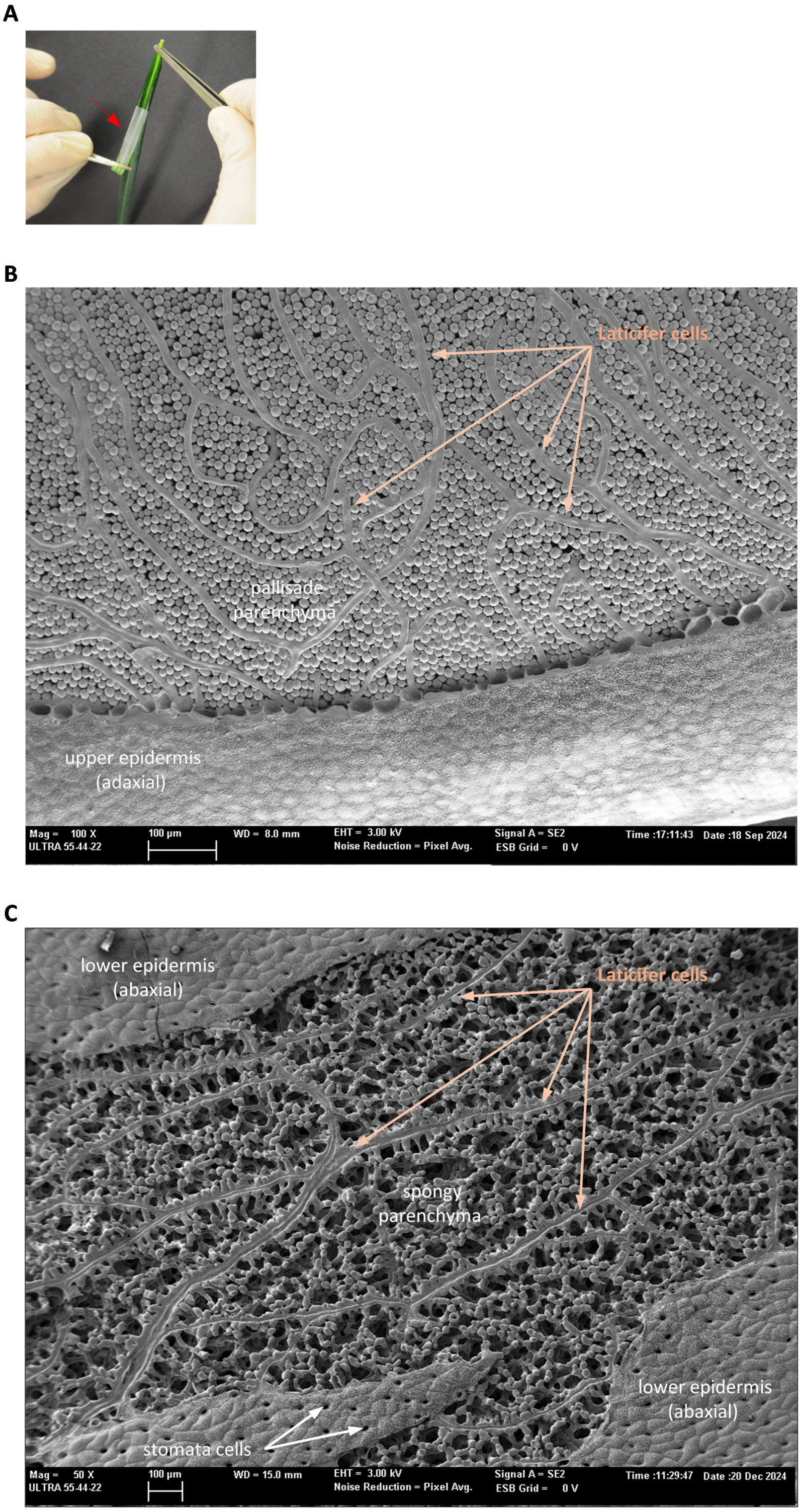

Supplemental Figure S1. Direct visualization of the way laticifers cells are distributed in leaves of *E. lathyris* plants as unveiled by Cryo-Scanning

electron microscopy (Cryo-SEM). (A) Epidermal peeling of *E. lathyri*s leaves. Manual adaxial peeling is shown, with a red arrow indicating the epidermal layer being released. (**B**) Representative Cryo-SEM image of an adaxial leaf sector upon partial peeling of the upper epidermal layer. The distribution of laticifer cells, which are sandwiched between epidermis and the palisade parenchyma tissues, is shown. (**C**) Cryo-SEM image of an abaxial leaf sector upon partial peeling of the lower epidermal layer. The distribution of laticifer cells, which are sandwiched between epidermis and the spongy parenchyma tissues, is shown. Some laticifer and stomata cells are indicated with arrows.

A

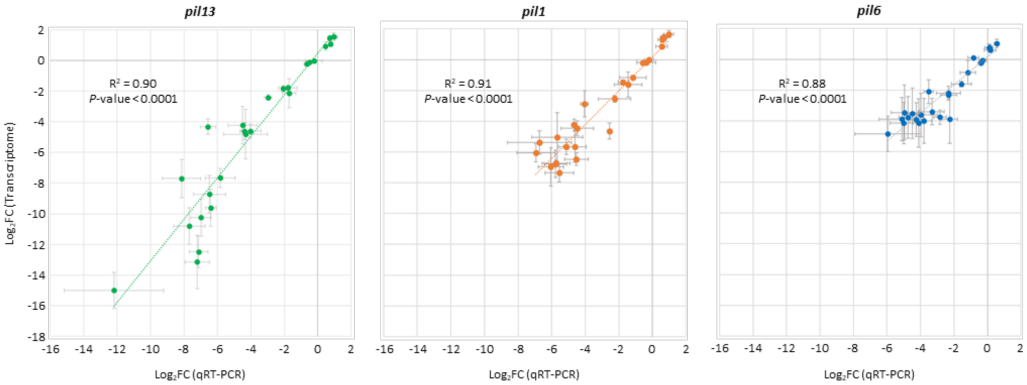

B

Gene	*pil13* qRT-PCR Log$_2$FC	qRT-PCR SD	Transcriptome Log$_2$FC	Transcriptome SD
Casp02478	-2.08	0.09	-1.84	0.20
Casp03411	-7.12	0.56	-12.48	1.03
Casp03871	-6.48	0.96	-8.73	1.22
Casp07584	-5.83	0.88	-7.66	0.62
Casp09460	-4.49	0.87	-4.22	1.22
Casp10131	-7.21	0.72	-13.14	1.74
Casp10582	0.97	0.21	1.54	0.26
Casp13615	-8.14	1.15	-7.70	1.26
Casp13745	-12.18	2.97	-15.00	1.18
Casp16493	-0.65	0.17	-0.22	0.15
Casp19002	-4.03	0.64	-4.60	0.46
Casp20664	-6.98	0.61	-10.24	1.21
Casp22112	0.72	0.09	1.45	0.21
Casp22590	-4.37	0.19	-4.61	0.54
Casp22865	-2.98	0.22	-2.41	0.20
Casp23831	-1.78	0.56	-1.79	0.17
Casp25391	-6.40	0.32	-9.62	1.20
Casp27543	-7.68	0.95	-10.81	1.20
Casp30083	0.76	0.13	1.07	0.15
Casp30750	-6.56	0.45	-4.32	0.51
Casp30753	-0.50	0.09	-0.12	0.14
Casp31220	-4.32	1.32	-4.81	1.62
Casp33047	0.46	0.11	0.92	0.28
Casp33262	-0.22	0.49	-0.03	0.13
Casp34410	-1.71	0.39	-2.13	0.94

Gene	*pil1* qRT-PCR Log$_2$FC	qRT-PCR SD	Transcriptome Log$_2$FC	Transcriptome SD
Casp02478	-1.76	0.29	-1.48	0.17
Casp03411	-5.74	0.84	-6.74	0.19
Casp03871	-2.56	0.12	-4.64	0.53
Casp07584	-4.53	0.71	-6.47	0.40
Casp09460	-4.04	0.23	-2.87	0.86
Casp10131	-4.62	0.68	-5.67	1.01
Casp10582	0.97	0.28	1.66	0.25
Casp13615	-6.04	0.75	-6.96	1.25
Casp13745	-6.94	1.13	-6.05	0.59
Casp16493	-0.57	0.04	-0.21	0.12
Casp19002	-4.66	0.16	-4.24	0.39
Casp20664	-6.72	1.90	-5.39	0.76
Casp22112	0.57	0.25	1.30	0.20
Casp22590	-4.48	0.97	-4.46	0.48
Casp22865	-2.25	0.96	-2.55	0.21
Casp23831	-1.18	0.80	-1.16	0.15
Casp25391	-5.13	0.47	-5.66	0.50
Casp27543	-5.55	0.85	-7.36	0.59
Casp30083	0.56	0.35	0.86	0.13
Casp30750	-5.77	0.67	-6.78	0.88
Casp30753	-0.20	0.27	0.00	0.11
Casp31220	-5.67	1.53	-5.03	1.58
Casp33047	0.67	0.21	1.44	0.19
Casp33262	-0.36	0.57	-0.17	0.11
Casp34410	-1.45	0.81	-1.62	0.81

Gene	*pil6* qRT-PCR Log$_2$FC	qRT-PCR SD	Transcriptome Log$_2$FC	Transcriptome SD
Casp02478	-2.32	0.47	-2.30	0.26
Casp03411	-4.11	0.32	-4.13	1.57
Casp03871	-5.00	0.84	-4.14	1.34
Casp07584	-4.25	0.41	-3.92	1.10
Casp09460	-3.52	0.28	-2.10	0.77
Casp10131	-3.79	0.45	-4.01	1.47
Casp10582	0.55	0.15	1.02	0.28
Casp13615	-4.96	0.79	-3.46	1.79
Casp13745	-5.95	1.96	-4.85	1.15
Casp16493	-0.39	0.18	-0.23	0.13
Casp19002	-3.31	0.73	-3.40	0.87
Casp20664	-5.12	1.36	-3.90	1.41
Casp22112	0.08	0.25	0.75	0.25
Casp22590	-2.84	0.38	-3.75	0.46
Casp22865	-2.33	0.72	-2.20	0.45
Casp23831	-1.56	0.60	-1.60	0.17
Casp25391	-3.98	0.98	-3.62	1.41
Casp27543	-4.75	0.54	-3.78	1.34
Casp30083	0.16	0.34	0.60	0.14
Casp30750	-4.48	0.39	-3.51	1.63
Casp30753	-0.29	0.21	-0.06	0.11
Casp31220	-2.26	0.46	-3.90	1.57
Casp33047	0.11	0.06	0.76	0.22
Casp33262	-0.85	0.32	0.11	0.11
Casp34410	-1.17	0.46	-0.86	0.77

Supplemental Figure S2. Laticifer-related transcriptome validation by qRT-PCR. (A). Linear regression analysis between log$_2$-fold change (*pil* mutant vs. wild-type plants) from randomly selected genes (n=25) analyzed by qRT-PCR vs. RNAseq. Mean ± SEM values were obtained from three biological replicates. (**B**)

Comparative gene expression levels (log$_2$ fold change) in *pil1*, *pil6* and *pil13* plants. Mean ± SEM values were obtained from three biological replicates.

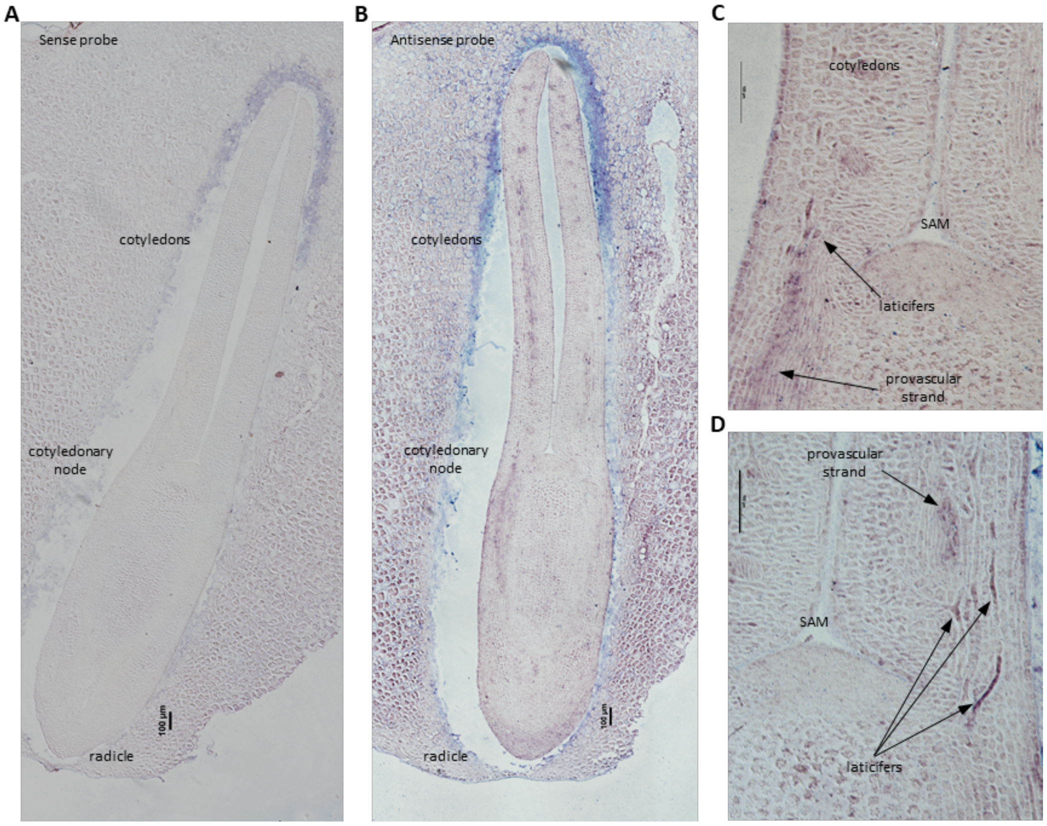

Supplemental Figure S3. In situ hybridization of *E. lathyris* embryos reveals strong expression of *Casp31220* in laticifer cells. A 192 bp fragment from Casp31220 was generated by PCR and cloned in pGEM-T. Digoxigenin-11-UTP (DIG) single stranded RNA probes were synthesized *in vitro* by the transcription of linearized plasmid template in the presence of DIG-UTP. Equal amount of sense and antisense DIG-labeled probes were used to hybridize paraffin-embedded embryos and embryos were subsequently incubated with anti-digoxigenin antibodies labeled with alkaline phosphatase. (**A**) *in situ* hybridization of embryos using a *Casp31220* sense DIG-labeled RNA probe. (**B**) *in situ* hybridization of embryos with a *Casp31220* antisense DIG-labeled RNA probe, which revealed hybridization signal associated to laticifer cells. (**C-D**) Magnified images around the shoot apical meristem (SAM) from two different embryos showing strong hybridization signal associated to laticifer cells located in the cotyledonary node.

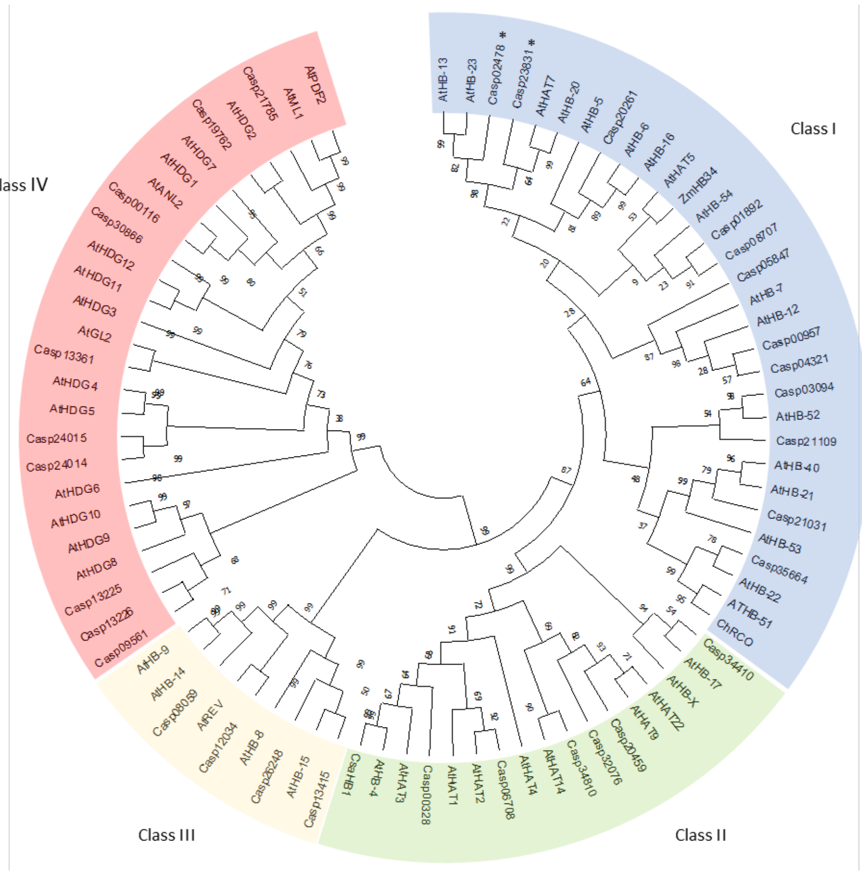

Supplemental Figure S4. Classification of *E. lathyris* HD-Zip proteins based on sequence homology. Circular phylogenetic tree of HD-Zip genes in Arabidopsis and *E. lathyis* in which subgroups in each HD-Zip subfamily (I, II, III, and IV) are indicated. Protein sequences were aligned by ClustalW. Each HD-Zip class is labeled. The evolutionary history was inferred using the UPGMA method (Sneath and Sokal. 1973. Numerical Taxonomy. Freeman, San Francisco). The optimal tree is shown. The percentage of replicate trees in which the associated taxa clustered together in the bootstrap test (2000 replicates) are shown next to the branches (Felsenstein. 1985. Confidence limits on phylogenies: An approach using the bootstrap. Evolution 39:783-791). The evolutionary distances were computed using the p-distance method (Nei and Kumar. 2000. Molecular Evolution and Phylogenetics. Oxford University Press, New York) and are in the units of the number of amino acid differences per site. This analysis involved 80 amino acid sequences. All ambiguous positions were removed for each sequence pair (pairwise deletion option). There were a total of 1115 positions in the final dataset. Evolutionary analyses were conducted in MEGA11 (Tamura , Stecher , and Kumar. 2021. MEGA 11: Molecular Evolutionary Genetics Analysis Version 11. Molecular Biology and Evolution.

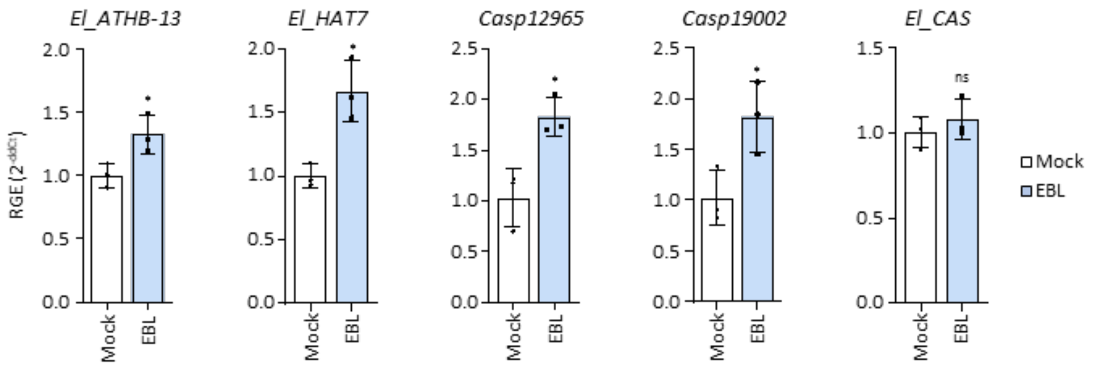

Supplemental Figure S5. Sustained enhanced expression of El_ATH7 and El_ATHB13 in EBL-treated leaves. Relative gene expression (RGE) of *El_HAT7, El_ATHB13, Casp12965, Casp19002* and *El_CAS* in leaves of *E. lathyris* plants at 15 days after application of 10 μM 24-epibrassinolide (EBL). Bars show the means ± SEM obtained from three biological replicates; asterisk indicate significant differences compared to control (mock) plant (*P*-value < 0.05; pairwise fixed reallocation randomization test).

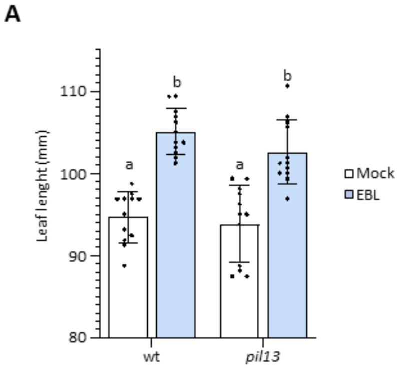

Supplemental Figure S6. Effect of Epibrassinolide (EBL) in promoting leaf growth enhancement in wild type and *pil13* plants. Leaf length was determined in newly formed leaves from control (mock) and EBL treated wild type and *pil13* plants at 15 days after treatment. Bars show the means ± SEM obtained from twelve biological replicates; asterisk indicate significant differences compared to control (mock) plants (** *P*-value < 0.01; Student's t-test).

A

Casp16883 (PRP1)

Nt_MARMRVSISFVLVLSLLATTGHSESSVVKHRKHIALPSLFPPLPIHPPPFSPLPPFRPPPLPLPPPPPSSNDCALATVQLVPCLIKV
HAFKKVDKNCCVVFNKCMLCIHFVPVVFLPAEIIAKCAAM_Ct

Casp16880 (PRP2)

Nt_MARNKVSMFFLMVVCVFGIAYTSNAENPVPKDSSSPPPDSPPPYIDCLEPIIGFGPCMAKVFGLLFVDQHCCDVFRKCEICM
YFIPQVILPLFAKEACGF_Ct

Casp16882 (PRP3)

Nt_MFPPFEPLLPPPLPPLFPPVEIPPPPPFLFPPFEPILPPPLPPLFPPRQIPPPPPPLPPLFPPIIHPPPPQQPSPEPPPPNQPPPIIPPP
SRPPPEQSPPDLPPPPSLFPPSPPSIFPPLPPLFPPPQQPPPVLPPPPPSLFPPSPPSIFPPLPPLFPPPQQPPLVLPPPPPSIFPPLFPPS
PILPPPPPPPPPPPPPSLFPPLPPLFPPVILPPPPQQPSPYQPPPLFPPPQFSPPVVPPPPKQPSPEQPPPVLPPPPSLFPPLPPILTPPP
LQPSPEEPPPILPPPPVEQPPPDQPPPILPPPPPPPSLFPPFVLPPPPQQPSPYQPPPVFPPLPPPPQQPSPDQPPPVLPPPPVLTPPP
QEPSPEQPPPILPPSPSLFPPLPPLFPPFILPPPPQQSPPDQPPPILSPPPSLFPPLPPLFPPPPPSLFPPPPEQPSPKQPPPILPPPPSLF
PPLPPLFPPPTEEPPPDQPPPPKQPPPILPPSPEQSPPMLPPPEYPPPTLPPPQQPPPTLPPLLPPLPPLFPPPLHPPPLPPLLPPPLTPI
FPPPLSPIFPPPLPPLPPLLPPPLFPPSPSPVPPPDCVLPTVQFSPCIIKVLVFKNIDEECCNVFRLCEICLDFKPKSVLPDFALTTCGFI_Ct

Ct_consensus aa sequence: -DC(N_9)C(N_{13})CC(N_5)C(N_2)C(N_{16})C-

B

		pil13	*pil1*	*pil6*	*Latex*	*wt*	*pil13*	*pil1*	*pil6*
Casp16883	*PRP1*				9795.3	179.0	0.0	1.9	9.1
Casp16880	*PRP2*				7555.3	49.2	0.0	0.7	3.5
Casp16882	*PRP3*				565.5	8.0	0.0	0.2	0.4

-5 log_2FC 5   0 TPM 250

C

Casp17273 (GRP)

Nt_MKKLYMTTMIFVALLLTASVLTQPQPKPAGPEPRSAGTPPQPKPAGPEPRSSSTPPQPKLAGPEPRSSGSGGAVVGGGGGGA
SAGGATVRKKNTKAKGGGTATGFGGGGTALGGRGTGFGGGGTGFGGGRGGGRRGGLVGGGAFVGGGARGHKNNASAPEISL
WRAVSNFGLVSFIYWCYF_Ct

D

		pil13	*pil1*	*pil6*	*Latex*	*wt*	*pil13*	*pil1*	*pil6*
Casp17273	*GRP*				2046.5	19.8	0.0	0.3	1.3

-5 log_2FC 5   0 TPM 250

Supplemental Figure S7. Amino acid sequence of cell wall-related proteins identified as DEGs associated to laticifer cells. (A) *Casp16883*, *Casp16880*, and *Casp16882* are tightly linked in the genome, presumably indicating diversification by gen duplication. We designated them as PRP1, PRP2, and PRP3, repectively, as they encode proline-rich proteins. The three PRPs share a higly conserved Ct consensus amino acid sequence domain rich in Cys residues

which follow a conserved spacing: -DC(N_9)C(N_{13})CC(N_5)C(N_2)C(N_{16})C-; where N represents any amino acid residue. (**B**) The expression of the three genes is highly compromised in *pil* mutants, with a complete supression in the case of *pil13* mutant, and the three genes are strongly expressed in laticifer cells as deduced from the analysis of TPM values counted on latex in comparison to whole leaf tissues. (**C**) *Casp17273* encodes a Glycine-Rich Protein (GRP), similar to cell wall GRP proteins from other plant species. (**D**) Gene expression of this GRP-encoding gene is strongly compromised in the three *pil* mutants, with a total suppresion in *pil13,* and is strongly expressed in laticifer cells as deduced from the latex transcriptome.

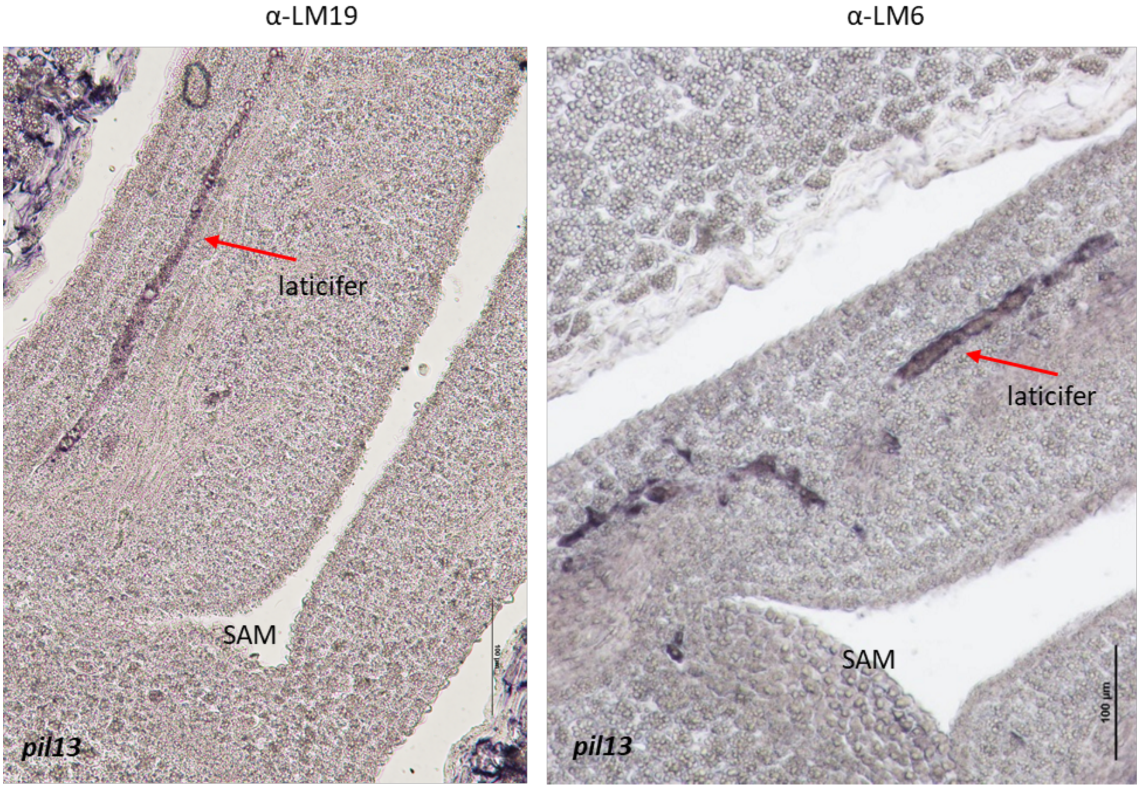

Supplemental Figure S8. Immunohistochemical localization of de-methylesterified HG, using α-LM19, and RG-I, using α-LM6, in laticifer cells present in embryos of *pil13* mutant. Laticifer cells are indicated with red arrows.

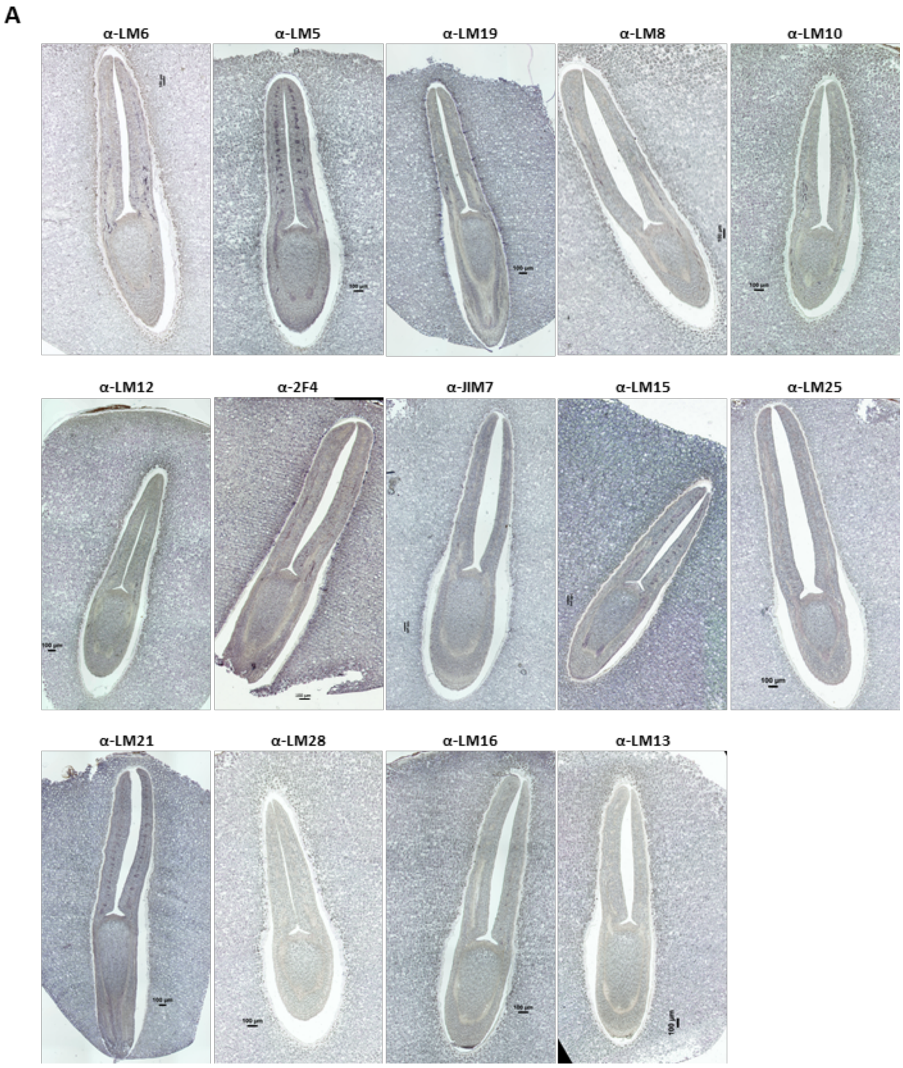

Supplemental Figure S9. Immunohistochemical analysis of whole of *E. lathyris* embryos as assayed with a range on antibodies recognizing distict pectin epitopes. Magnified images around the shoot apical meristem images are shown in Figure 5E.

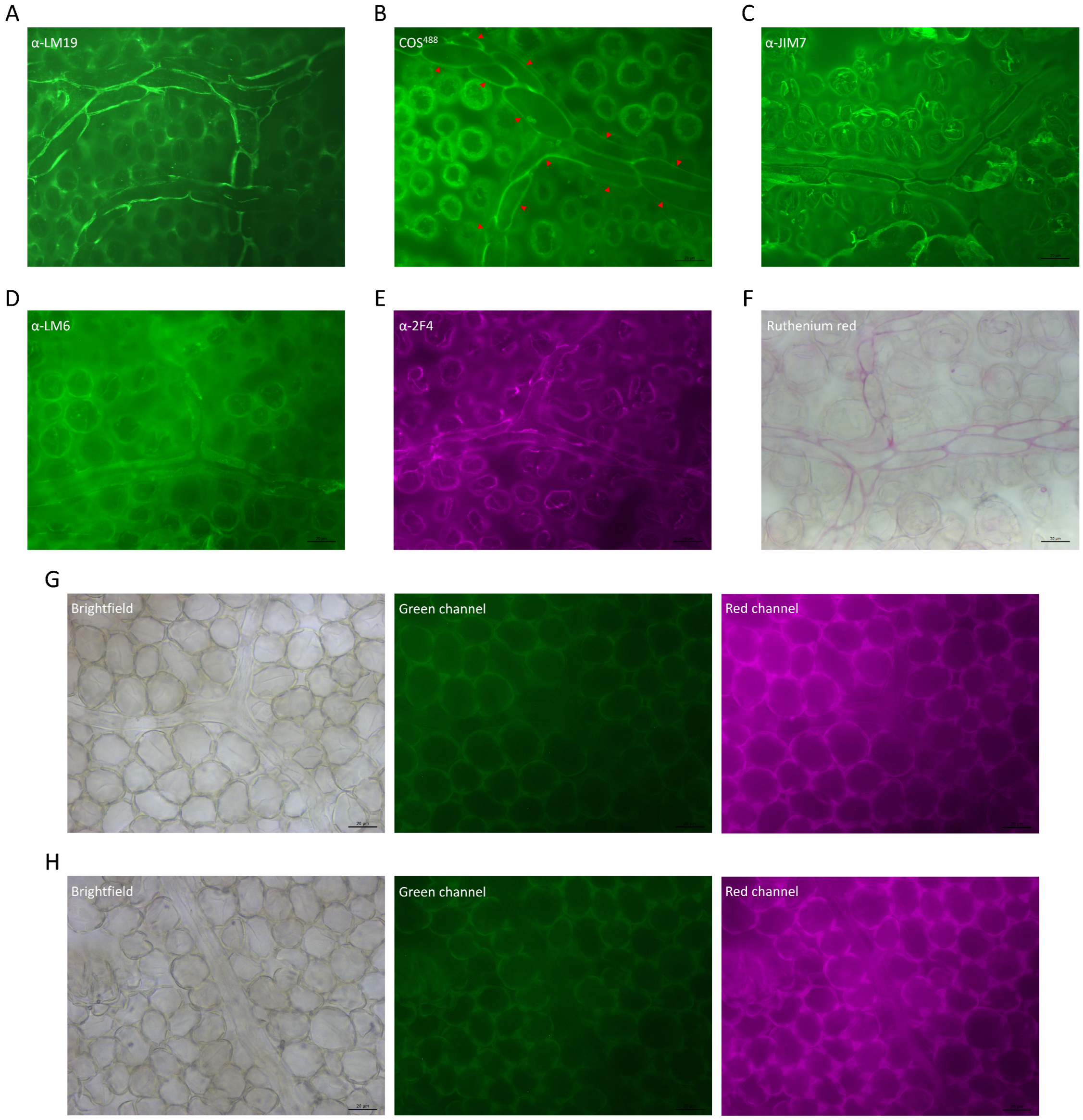

Supplemental Figure S10. Deposition of pectin polymers in laticifer cells in *E. lathyris* leaves. (A). Localization of de-methylated HG accumulation using α-LM19. (**B**). COS^{488} labeling of de-methylated HG. Red arrows indicate the discrete contacting ovals of de-esterified HG polymers. (**C**). Localization of methylated HG accumulation using α-JIM7. (**D**). Localization of RG-I accumulation using α-LM6. (**E**). Localization of de-methylated HG crosslinked to Ca^{2+} using α-2F4. (**F**) Ruthenium red staining. **(G).** Secondary-antibody-only control showing no signal associated with laticifer cells. **(H).** Autofluorescence control.
