# Supplementary material for: A HAT7/ATHB13/brassinosteroid signaling module mediates growth of laticifer cells in Euphorbia lathyris
Source: Plant Cell. 2026 Oct 17;38(10):koag289. doi: 10.1093/plcell/koag289 (PMC13711675; doi:10.1093/plcell/koag289)
Supplement: Supplementary file 1 [file koag289_supplementary_data.zip › Supplemental Tables.pdf]

SUPPLEMENTAL TABLES 2 to 4

Supplemental Table 2. Primer sequences used for qRT-PCR gene expression analysis

Gen	Description	Name			Sequence	
Casp35842	Ubiquitina	UBQ	Fw	5`	GCAGAAAGTGTTATGCTCGCC	3`
			Rv	5`	TCTTGCGACAGTTAACAGCCC	3`
Casp03973	Elongation factor	EFACT	Fw	5`	TAAGGATGATCCAGCCAAGG	3`
			Rv	5`	TTCTCAAGCTCCTTCCCAGA	3`
Casp30753	Cicloartenol synthase	CAS	Fw	5`	TGAAGGGGCTAATGATGGAG	3`
			Rv	5`	ACCACTCAAATGCTCCAAGC	3`
Casp22590		EH	Fw	5`	ACAGGCTTTACCGGTGGAATC	3`
			Rv	5`	CAGCTCCCATGTACGGTCAA	3`
Casp03411		PE	Fw	5`	GGTAGCCTCAACGTGGTGGA	3`
			Rv	5`	CGCACCCATACGTCTCTTAGG	3`
Casp25391		DHDDS	Fw	5`	CCGGCTCTTCAGCTCTTATGA	3`
			Rv	5`	TTATGCCGAGTTCCCAACAAT	3`
Casp02478	HD-ZIP I	ATHB13	Fw	5`	GGAATGGCTTTCTTCCCTGC	3`
			Rv	5`	ATGCCACCCCGTTGAAATCT	3`
Casp23831	HD-ZIP I	HAT7	Fw	5`	TTGTCCTCCCCACCTTTTTC	3`
			Rv	5`	TTTCTCAACCCCAGAGAAGG	3`
Casp12965			Fw	5`	TTTTGGGGTTGTTAGCCTTG	3`
			Rv	5`	ACCTGGGTGATGAACAGCTT	3`
Casp07584			Fw	5`	CTTCACCGTTCTGGAAGCTC	3`
			Rv	5`	TCTTCCTTCTTCCCCCTTGT	3`
Casp30083			Fw	5`	TTGGGAGAGGCTACGGAAGA	3`
			Rv	5`	TCAGCAGAAGCATCAAGGCA	3`
Casp19002			Fw	5`	GCACAATGGCAACCTTGGAG	3`
			Rv	5`	GACAGTCGTGGTGCAGGTAA	3`
Casp18847	Cdc10-dependent transcript 1	CDT1a	Fw	5`	TCCGGCGAAGTATGAGATGC	3`
			Rv	5`	TTCAACCGCAGTAGCCGAAT	3`
Casp28095	Cyclin	CYCB1	Fw	5`	GATGTAGCCGCCGAGAATCA	3`
			Rv	5`	CCGCCTACCTTGTTCACCTT	3`
Casp03871	Cellulose synthase	CESA	Fw	5`	TCATCAACCCATTGTCCAGA	3`
			Rv	5`	GAATTCATGGCACCAGCTTT	3`
Casp03411	Pectinesterase	PME	Fw	5`	GCTGGTTGGGACAGATGGAA	3`
			Rv	5`	AGCCAGGCCAATTCACTCTC	3`
Casp16880	Proline-rich protein	PRP2	Fw	5`	ATGCTGAAAACCCAGTCCCC	3`
			Rv	5`	CCACAAGCTTCTTTGGCAAAC	3`
Casp16882	Proline-rich protein	PRP3	Fw	5`	TCCAGTTCCACCTCCTGATT	3`
			Rv	5`	CAAATCTCGCACAATCGAAA	3`
Casp20333	Membrane bound O-acyl transferase	MBOAT	Fw	5`	TCGTGTAGTCGGGAGGAAGT	3`
			Rv	5`	CACGAGATCAGTGGAGGCAA	3`
Casp01943	β-1,4-Galactan synthase	GalS1	Fw	5`	TGGAACTGTGGCCGTAGAAC	3`
			Rv	5`	AGGTACCCAGTTGCAGCATC	3`

Casp09460		Fw	5`	GGTGTTTGATTCAGGCCAAG	3`
		Rv	5`	GAGCGGGTAAATTGAGATCC	3`
Casp10131		Fw	5`	CACGATTGCAACACTGCTCT	3`
		Rv	5`	TTAAAACTCGCCAGCTTGGT	3`
Casp10582		Fw	5`	GCAAGACAAGCAATCTGCCC	3`
		Rv	5`	TTCCACGCATTCTGCAGAGT	3`
Casp13615		Fw	5`	GGTTTGTGGTCTCCAGAGGA	3`
		Rv	5`	CCACGTTTCAGGTCTGGTCT	3`
Casp13745		Fw	5`	TTCAAGTCGCGGTCCAAACT	3`
		Rv	5`	TTGATGGAGAAACGGCACCA	3`
Casp16493		Fw	5`	TTGGATCTCGCTTCTGGTCT	3`
		Rv	5`	CCAGCCGAACTCATAGAAGC	3`
Casp20664	BUT2	Fw	5`	AAGCAGGTTGAGGTTGTTGG	3`
		Rv	5`	TGGCTCCCAAGCTGAAATAC	3`
Casp22112		Fw	5`	GCCCTCGAACCTGCTACTTT	3`
		Rv	5`	ACCGGCCTTTTGTATACGCT	3`
Casp22865	BUT1	Fw	5`	TCTCACTCATGCTTGCTTGG	3`
		Rv	5`	CAGCGACCCATAACAAATCC	3`
Casp27543		Fw	5`	TGATGCTAGAGTTGGCGATG	3`
		Rv	5`	GAGGGCGCTTTTCTTTCTTT	3`
Casp30750	LAS	Fw	5`	TGGGATGTTGTGTTTGCTGT	3`
		Rv	5`	TGGAGTTGAAAAAGGCCATC	3`
Casp31220		Fw	5`	ATGGCAGTTTCGTTGTTTAG	3`
		Rv	5`	TCATGGCATATACAACTTGT	3`
Casp33047		Fw	5`	TCCCCTTCACACTCATCCCA	3`
		Rv	5`	CCCGTCATTCCAGGCATACA	3`
Casp33262		Fw	5`	TCCCTGCCAAACTGTTAAGG	3`
		Rv	5`	CCAGGGCTCCTTTTTCTTTC	3`
Casp34410		Fw	5`	CAACGGGGACACATTGTAAG	3`
		Rv	5`	GCTTAGGGCAATGAAAGTGG	3`

Supplemental Table 3. Primer sequences used to obtain the specific amplicon for VIGS experiment

Gen	Description	Name			Sequence	
Casp02478	HD-ZIP I	ATHB13	Fw	5`	cacttacccgagttaacgccAGCCATGGCCAAAATCCAG	3`
			Rv	5`	gtttaatgtcttcgggacatAAGAAACGGAGGGGTCTTG	3`
Casp23831	HD-ZIP I	HAT7	Fw	5`	cacttacccgagttaacgccGTCATTGATGTTGGCCTTATTG	3`
			Rv	5`	gtttaatgtcttcgggacatTAAGCAGTTGGAGAAAGATTATG	3`

Supplemental Table 4. Primer sequences used to obtain the specific amplicon for RNA *in situ* hybridization.

Gen			Sequence	
Casp31220	Fw	5`	ATGGCAGTTTCGTTGTTTAG	3`
	Rv	5`	TCATGGCATATACAACTTGT	3`
